# Photoperiod-Dependent Expression of MicroRNA in *Drosophila*

**DOI:** 10.3390/ijms23094935

**Published:** 2022-04-29

**Authors:** Mirko Pegoraro, Bettina Fishman, Valeria Zonato, Georgios Zouganelis, Amanda Francis, Charalambos P. Kyriacou, Eran Tauber

**Affiliations:** 1 School of Biological and Environmental Sciences, Liverpool John Moores University, Liverpool L3 3AF, UK; m.pegoraro@ljmu.ac.uk (M.P.); a.j_francis@hotmail.co.uk (A.F.); 2 Department of Evolutionary & Environmental Biology, Institute of Evolution, University of Haifa, Haifa 3498838, Israel; bfishman@univ.haifa.ac.il; 3 Department of Genetics and Genome Biology, University of Leicester, Leicester LE1 7RH, UK; vz12@leicester.ac.uk (V.Z.); cpk@leicester.ac.uk (C.P.K.); 4 School of Human Sciences, University of Derby, Derby DE22 1GB, UK; g.zouganelis@derby.ac.uk

**Keywords:** diapause, *Drosophila*, microRNA, photoperiodism, RNA immunoprecipitation, seasonal timing

## Abstract

Like many other insects in temperate regions, *Drosophila melanogaster* exploits the photoperiod shortening that occurs during the autumn as an important cue to trigger a seasonal response. Flies survive the winter by entering a state of reproductive arrest (diapause), which drives the relocation of resources from reproduction to survival. Here, we profiled the expression of microRNA (miRNA) in long and short photoperiods and identified seven differentially expressed miRNAs (*dme-mir-2b*, *dme-mir-11*, *dme-mir-34*, *dme-mir-274*, *dme-mir-184*, dme-mir-184*, and *dme-mir-285*). Misexpression of *dme-mir-2b*, *dme-mir-184*, and *dme-mir-274* in pigment-dispersing, factor-expressing neurons largely disrupted the normal photoperiodic response, suggesting that these miRNAs play functional roles in photoperiodic timing. We also analyzed the targets of photoperiodic miRNA by both computational predication and by Argonaute-1-mediated immunoprecipitation of long- and short-day RNA samples. Together with global transcriptome profiling, our results expand existing data on other *Drosophila* species, identifying genes and pathways that are differentially regulated in different photoperiods and reproductive status. Our data suggest that post-transcriptional regulation by miRNA is an important facet of photoperiodic timing.

## 1. Introduction

Adaptation to seasonal fluctuations in the environment drives some of the most incredible changes in animal behavior and physiology [1]. Examples of common strategies for escaping unfavorable seasons include bird migration, insect diapause, and mammalian hibernation. The accurate anticipation of the incoming season is vital for development, reproduction, and fitness, and requires a seasonal timer [2,3] that is often based on photoperiod measurement. The molecular details of this photoperiodic timer are still elusive, despite decades of intense research [2,4]. Recently, however, significant progress has been made [5,6,7,8]. 

In *Drosophila melanogaster*, the seasonal response is triggered by the short autumnal photoperiod and decreasing temperatures [9]. The response is manifested as an arrest of ovarian maturation in a previtellogenic state that is characteristic of a reproductive diapause. The photoperiodic response in *D. melanogaster* is rather shallow and strain-specific and is largely masked by the temperature cycles [7]. A recent study [10] suggested that the photoperiodic response of flies in the laboratory could be greatly enhanced by adopting a semi-natural lighting regime, as opposed to the rectangular light–dark cycles which are commonly used. The photoperiodic response of *Drosophila* is also increased when the flies are starved [5]. 

The mechanism of photoperiodic timing is still obscure, although, recent evidence points to the rather old internal coincident model suggested by Pittendrigh [11]. In this model, the phase relationship between two circadian oscillators that are, respectively, driven by scotophase (night) and photophase (light) is used to encode the photoperiod. The circadian neuronal network in *Drosophila* consists of two distinct groups of clock neurons, each oscillating at a different phase (M-cells and E-cells) [12], which could also serve as a photoperiodic timer. M-cells produce pigment-dispersing factor (PDF) that is used to synchronize E-cells (via the PDF receptor) [12], while projections from M-cells and from insulin-producing cells (IPCs), which are essential for diapause activation, converge in the dorsal protocerebrum [13]. Remarkably, the loss of PDF in *Pdf^01^* mutants is sufficient to abolish the photoperiodic response [5]. 

Control of the diapause program in *D. melanogaster* involves insulin-like peptides. These are secreted from the IPCs in the mid-brain, stimulating the *corpora allatum* (CA; a subunit of the ring gland, a tripartite endocrine organ) to produce the juvenile hormone (JH). JH promotes the synthesis of yolk proteins in the fat bodies [14]. In the presence of JH, the ovaries are induced to produce ecdysteroid (Edc), which promotes yolk protein upload and vitellogenesis. The shutdown of JH and Edc determines the arrest of ovarian maturation in a previtellogenic state that is characteristic of *D. melanogaster* diapause [15,16]. 

MicroRNAs (miRNAs) are short non-coding RNAs which bind to the target 3′ untranslated region (UTR) of mRNA molecules and regulate expression of the encoded gene at the translational level [17]. Active and mature miRNAs are 17–24-base-pair-long single-stranded RNA molecules that are expressed in eukaryotic cells and modulate the translation or stability of target messenger RNAs (mRNA). MiRNAs are generated by cleavage of their pri-mRNA precursors by the endoribonuclease Drosha, followed by cleavage by Dicer in the cytoplasm. The miRNA is loaded into the RISC complex that contain Argonaute-1 (AGO-1), and then binds to target transcripts, particularly their 3′UTR regions. Binding of the miRNA to target region leads to either cleavage of the target transcript or inhibition of their translation. 

Post-transcriptional regulation by miRNA has been implicated in a broad range of processes, including the circadian system [18]. Recent studies in plants revealed that miRNAs are also involved in the photoperiodic timing of flowering [19,20], but no similar role in animal photoperiodism has been reported to date. Here, we report a global expression profiling of RNA extracted from diapausing and reproductive females, as well as from females exposed to different photoperiods, and identify differentially expressed genes (DEGs) and miRNA (DEM) and demonstrate their functional role in photoperiodism.

## 2. Results

### 2.1. Differential Gene Expression

**Figure 1:**
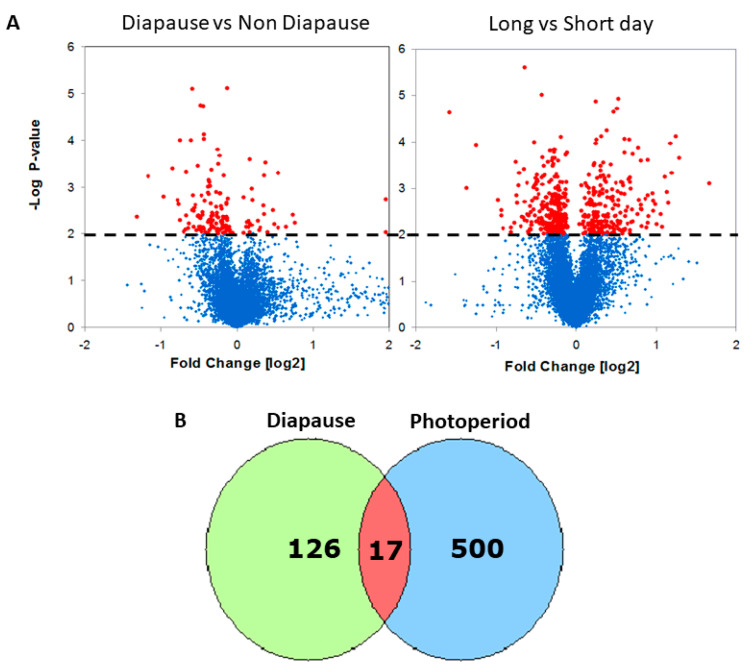
Transcriptional response to diapause and photoperiod in *Drosophila.* (**A**) Volcano plots depicting the statistical significance (y axis) vs. the fold change (x axis) of differential expression in female heads (red: *p* < 0.01; blue: NS). In the diapause experiment (**left**), positive fold changes represent upregulation in diapause. In the photoperiodic experiment (**right**), positive fold changes represent upregulation in long-day readings. (**B**) Overlapping Venn diagrams of photoperiodic and diapause DEGs.

To identify genes associated with diapause, we exposed the females to a critical day-length photoperiod (14 h), which by definition, induces diapause in 50% of the females, and compared gene expression in the heads of diapausing and non-diapausing females. We identified 143 DEGs (*p* < 0.01; Figure 1, Table S1). Significantly enriched gene ontology (GO) biological function among the upregulated genes in diapause was “proteolysis”, while in downregulated gene-enriched GO functions included “protein binding”, “actin cytoskeleton organization”, and “oogenesis” (Benjamini-Hochberg FDR *q* < 0.2, Table S2). 

To identify genes associated with photoperiodic diapause induction, we carried out a sperate experiment where we compared gene expression in females maintained in either long or short photoperiods. We identified 517 DEGs (*p* < 0.01; Figure 1, Table S1). Among the top hits were *Hsp70* proteins which were previously implicated in insect diapause [21]. Other interesting genes identified were *vrille* (*vri*) [22] and *shaggy* (*sgg*) [23], clock genes that may serve as a link between the circadian and photoperiodic clocks. 

Over-represented GO biological processes among the upregulated photoperiodic DEGs included “fatty-acyl-CoA binding” and “oxidation-reduction process”. Among the downregulated photoperiodic DEG, enriched functions included “fatty-acyl-CoA binding”, “fatty acid beta-oxidation”, and “cellular response to starvation” (Table S2).

The intersect of the two lists reveals 17 transcripts that may serve as candidate loci for linking the photoperiodic timer with diapause induction (Figure 1, Table S3). These included genes encoding *yolk proteins 2-3*, *deoxyribonuclease II*, *CG3829*, *CG1CG1*, *G3829*, *CG10516*, *CR17875* (heme-binding), and *Obp99a*. The gene encoding another odorant-binding protein in the list (*Obp99b*; Table S1) is known to be regulated by Juvenile Hormone (JH) [6,24].

### 2.2. miRNA Expression

**Figure 2:**
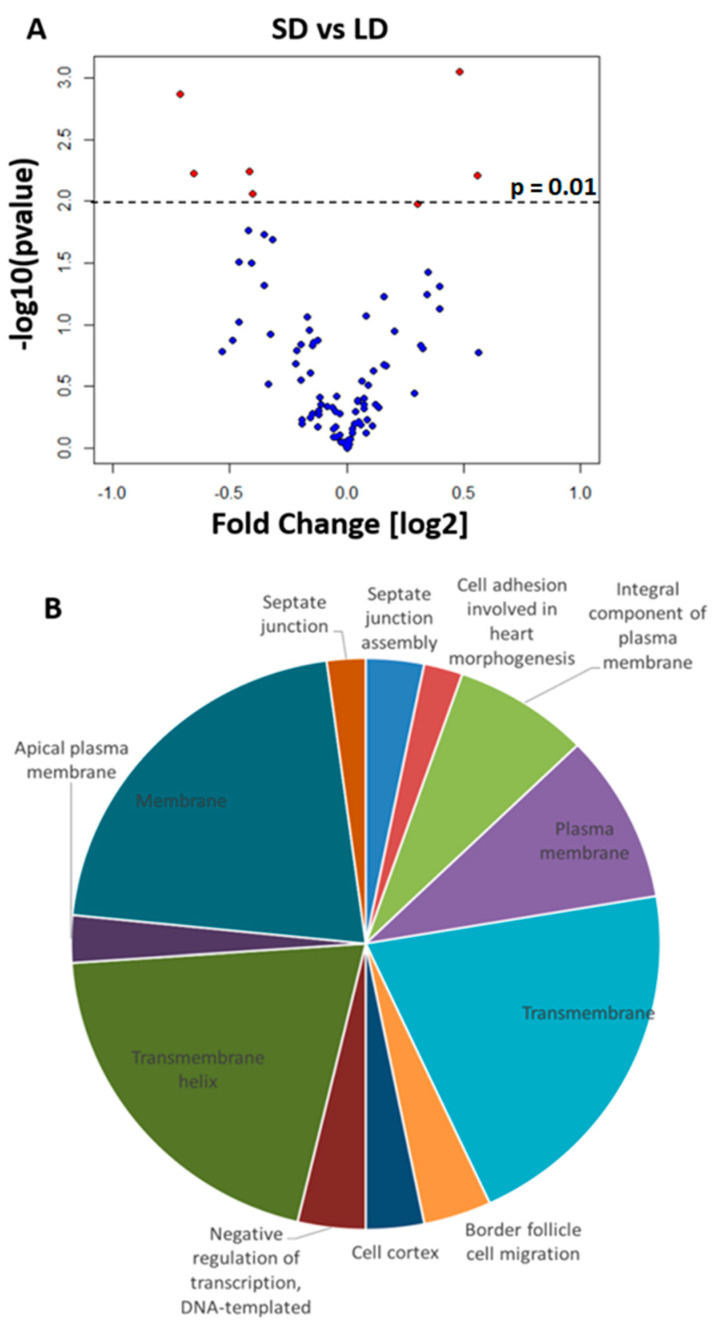
Differences in miRNA expression. (**A)** Volcano plot showing differentially expressed miRNA in female heads (*p* < 0.01, red; *dme*-mir184* p = 0.0104) exposed to long-day (LD) and short-day (SD) conditions. Positive fold changes indicate upregulation in short-day readings. (**B**) Pie chart showing enriched biological functions (DAVID, Benjamini *p* < 0.05) of the 20 most probable targets for each DEM. The size of the pie section is proportional to the number of genes for that biological function. SD: short day. LD: long day.

We found substantial photoperiodic difference in expression and therefore we asked whether post transcriptional microRNA mediated control of expression could also contribute to the flies’ photoperiodic response. We measured the expression of miRNA in fly heads exposed to either long or short photoperiods using microarrays. We detected seven differentially expressed miRNA (DEMs) that included *dme-mir-2b*, *dme-mir-11*, *dme-mir-34*, *dme-mir-274*, *dme-mir-184*, *dme-mir-285* (all at *p* < 0.01), and dme-mir-184* (*p* = 0.01, Figure 2). 

To test for biological function enrichment among the miRNA targets, we analyzed the gene ontologies of the top 20 genes targeted by the DEMs (Table S4). We found significant enrichment of processes such as “septate junction”, “membrane activity”, and “negative regulation of transcription”. A number of genes were also targeted by more than one miRNA (Table S4); although, their function in diapause or a photoperiodic response has yet to be elucidated. Overall, these results suggested that miRNAs may play an important regulatory role in the fly photoperiodic response

### 2.3. Network Analysis

**Figure 3:**
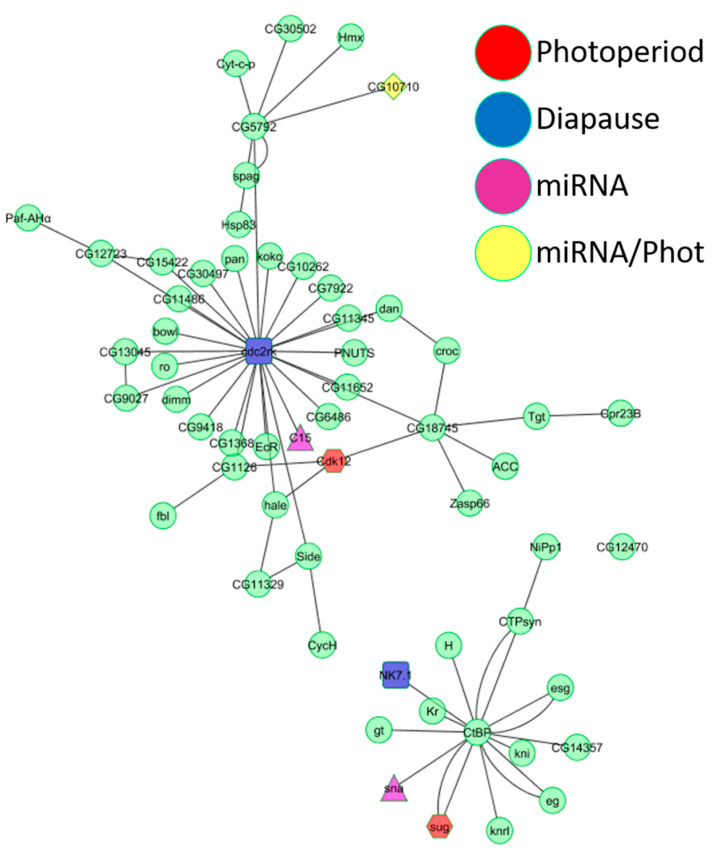
Intersection of yeast two-hybrid interaction networks. The intersection of the three yeast two-hybrid interaction networks (diapause, photoperiodic, and miRNA) resulted in two interaction pathways. The largest interaction pathway (**top**) has at its center at *cdc2-related-kinase*, a diapause DEG (Table S1). The second interaction pathway has at its center at the gene for the *C-terminal-binding protein*. Red nodes are photoperiodic DEGs, light blue nodes are diapause DEGs, purple nodes are DEMs targets, and yellow nodes are both DEMs targets and photoperiodic DEGs.

To identify the pathways associated with the photoperiodic response, we generated a network of protein–protein interactions [25,26] combining each of the DEG lists (Diapause and photoperiodic) with the top 20 target genes of each DEM. As shown below, the network analysis identified biological functions not immediately evident from the microarray results (Figure 3). 

The photoperiodic network consisted of GO biological functions such as “rRNA methylation”, “histone phosphorylation”, “gene silencing by miRNA”, and “regulation of circadian sleep/wake” (Table S5). “Entrainment of the circadian clock”, “regulation of circadian rhythm”, “response to heat”, “visual behavior”, and “JAK-STAT cascade” were represented in both the miRNA and photoperiodic networks (Table S5). The diapause network contained just one unique GO biological function (“fat body development”) but shared many biological functions with both the photoperiodic network (“tRNA methylation”, “regulation of translational initiation”, and “phospholipid biosynthetic process”) and the miRNA network (“ecdysone receptor-mediated signaling pathway”, “positive regulation of hormone secretion”, and “rhodopsin-mediated signaling pathway”). A unique biological function of the miRNA network was “mitochondrial electron transport” (Table S5). We generated another network intersecting the photoperiodic, diapause, and miRNA datasets, which revealed two main pathways (Figure 3). The central node of the largest graph was *cdc2-related-kinase*, encoded by a diapause DEG. In the second graph, a photoperiodic DEG, a diapause DEG and a DEM all interacted with the central node (the *C-terminal-binding protein* gene), suggesting that a minute change in expression of just a few genes can have a cascading effect on a larger gene network. The biological functions represented in the larger interaction network were “fatty acid biosynthetic process”, “positive regulation of hormone secretion”, “regulation of transcription”, “regulation of circadian sleep/wake cycle”, “positive regulation of apoptosis”, “ecdysone receptor-mediated signaling”, “determination of adult lifespan”, and “nuclear mRNA splicing” (Figure 3). The biological functions in the second network were “regulation of transcription”, “olfactory behavior”, and “Notch signaling pathway”. Biological functions, such as “regulation of alternative mRNA splicing”, “chromatin silencing”, “regulation of Notch signaling pathway”, “courtship behavior”, and “phototransduction”, were shared by all three microarray experiments (Table S5). 

### 2.4. miRNA Misexpression

**Figure 4:**
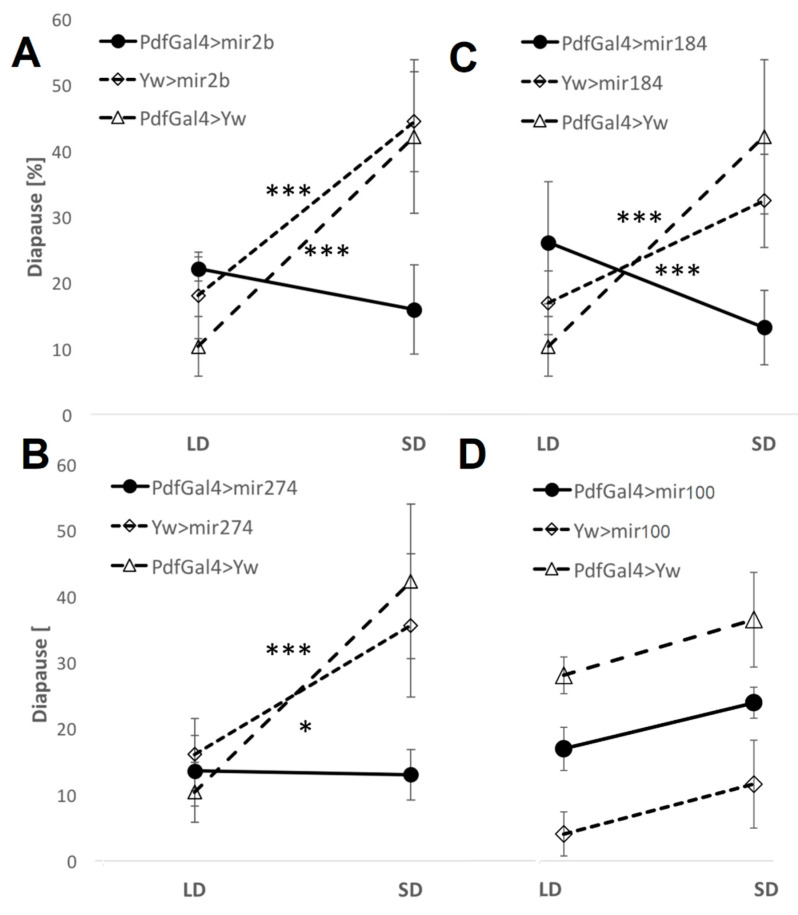
miRNA overexpression causes a loss of photoperiodic response. Average diapause [%] in flies overexpressing (**A**) *dme-mir-2b*, (**B**) *dme-mir-184*, (**C**) *dme-mir-274*, and *dme-mir-100* (**D**), in PDF-expressing cells. Error bars represent SEM. Significant Phot:Gen interactions [*p* < 0.001 (***), *p* < 0.05 (*)] indicate a different photoperiodic behavior between overexpressing lines and controls. *dme-mir-100* is not a photoperiodic miRNA, and the experiment serves as a negative control.

To explore the functional roles of DEMs in the photoperiodic response, we induced the overexpression of *dme-mir-2b*, *dme-mir-184,* and *dme-mir-274* using UAS transgenes driven by strains carrying *Act*-Gal4 (ubiquitous expression) or *pdf*-Gal4 (in *pdf*-expressing neurons) and tested for any changes in photoperiodic diapause. We compared changes in photoperiodic responses between control (i.e., flies carrying a single transgene) and overexpressing (i.e., flies with both the UAS and the GAL4 transgenes) lines by fitting a generalized ANOVA linear model. A significant photoperiod × genotype interaction indicates a significant effect of the misexpression. The overexpression induced by *Act*-Gal4 resulted in almost total lethality (data not shown), while overexpression in PDF-expressing neurons resulted in a loss of the photoperiodic response (Figure 4). Misexpression of *mir274* abolished the difference between long- and short-day flies (compared with UAS-*mir274* control, χ^2^_1,16_ = 5.51, *p* < 0.05 and *pdf*-Gal4 χ^2^_1,16_ = 11.28, *p* < 0.001), indicating a substantial reduction in short-day diapause compared with controls (Figure 4). Overexpression of *dme-mir-2b* resulted in a similar outcome (*Pdf*-Gal4 > mir2b vs. UAS-mir2b, χ^2^_1,18_ = 12.26, *p* < 0.001; *Pdf*-Gal4 > mir2b vs. *Pdf*-Gal4, χ^2^_1,16_ = 17.05, *p* < 0.001), while in the case of *mir184*, the short-day reduction was accompanied by an increased percentage of long-day diapause (Figure 4; *Pdf*-Gal4 > mir184 vs. UAS-*mir184*, χ^2^_1,19_ = 11.58, *p* < 0.001; and vs. *Pdf*-Gal4 > Yw, χ^2^_1,18_ = 25.52, *p* < 0.001). 

### 2.5. AGO-1 Immunoprecipitation

The availability of highly specific antibodies against AGO-1 allows for the isolation of functional Ago-miRNA-mRNA complexes. To identify the transcripts that are under miRNA regulation, we used AGO-1 immunoprecipitation of RNA samples derived from flies maintained in either long- or short-day conditions. 

We sequenced the associated RNA, and tested for transcripts enriched in either long- or short-day samples. We found 643 genes whose transcripts were significantly enriched in long-day immunoprecipitates, and 68 genes that were enriched in short-day immunoprecipitates (q < 0.05, Table S6). Among the long-day genes there were 62 over-represented biological functions (FDR < 0.05; Figure S1), including “alternative splicing”, “regulation of transcription”, “zinc-finger”, “transmembrane activity”, “septate junction”, and “eye development”. In contrast, in the short-day gene list, “ether lipid metabolism” was the only marginally significant term (FDR = 0.058; Figure S1). A number of targeted genes were particularly interesting. The long-day list included genes such as *AGO1*, *ecdysone receptor* (*EcR*), and circadian clock genes, such as *clock* (*Clk*), *pdp1*, and *vri* (Table S6). Genes of the insulin pathway were also enriched under long-day conditions (i.e., *InR* and *Ilp6*; Table S6). In addition, some predicted DEM targets were present in the long-day list (i.e., *comm2*, *Tsf2*, *Hs3st*-A, *sinu*, *CG43780*, Nrx-IV, *Pcl,* and *trbl*) and in the short-day list (*sinu*) (Table S6). Interestingly, insulin-like receptor (*InR*) was one of the 51 enriched genes common to both the short- and long-day lists (Table S7). 

## 3. Discussion

This study aimed to test the role of miRNA in the photoperiodic response of *Drosophila*. We identified seven miRNAs that were differentially expressed between long and short photoperiods (i.e., dme-mir-2b, dme-mir-11, dme-mir-34, dme-mir-274, dme-mir-184, dme-mir-184*, dme-mir-285). The roles and functions of some of these miRNAs in *Drosophila* are not clear yet (such as *dme-mir-2b, dme-mir-274, dme-mir-285*). However, the biological processes affected by *dme-mir-11, dme-mir-34*, and *dme-mir-184* show an intriguing link to photoperiodism and diapause. *dme-mir-11*, for example, was implicated in regulating apoptosis during embryonic development [27] and in regulating pupal size during metamorphosis [28]. Interestingly, *dme-mir-34* plays an important role in early neuronal differentiation and ageing [29,30], as well as in ecdysone signaling [31]. *dme-mir-184* is involved in oogenesis, development, and ovary morphogenesis [32,33,34], while the daily expression of this miRNA shows circadian oscillation [35]. Overexpressing *dme-mir-2b, dme-mir-184,* and *dme-mir-274* using *Act*-Gal4, a driver inducing spatially and temporally broad expression, resulted in almost total lethality, suggesting central roles for these three miRNAs during embryogenesis/development. Remarkably, overexpression of these miRNAs in PDF-expressing cells resulted in a loss of photoperiodic response, albeit in different ways. Since the PDF-expressing neurons are known to be involved in the circadian clock, it is possible that the photoperiodic impairment was due to loss of circadian function, as these two processes might be linked [5]. This interpretation cannot be excluded, as circadian function was not analyzed in these flies. 

Our microarray experiments suggested that both *dme*-*mir-2b* and *dme-mir-274* are expressed at higher levels in long days than in short days. Consistent with this observation, overexpression resulted in a substantial reduction in diapause in short days, when there is normally a lower level of these miRNA (as compared with long days). *dme-mir184* is highly expressed in short days, with its overexpression resulting in a reduction in short-day diapause, accompanied by an increase in long-day diapause and a reversal of the normal photoperiodic response. These data suggest a possible causative role of these miRNAs in the photoperiodic response. 

Recently, dme-*mir-210* was implicated in mediating clock function and light perception, possibly affecting the arborization of the ventral lateral neurons (LNv, pdf+ cells) [5,36]. It was suggested [5] that light information reaches the IPCs via PDF-expressing cells. As such, it is possible that miRNA controlling expression in these cells are either integral part of the photoperiodic time measurement or have a cascade effect on it. Our miRNA microarray experiment suggests that there are more miRNAs overexpressed in long-day than in short-day flies (Figure 2). Consistently with this observation, the long-day AGO-1 immunoprecipitates contained almost 10 times more enriched genes than did the short-day immunoprecipitates (643 vs. 68; Table S6). Interestingly, “septate junction” and “membrane activity” functions were enriched both amongst the DEMs targets and in the long-day AGO-1 immunoprecipitated genes. 

In addition, we identified 143 genes differentially expressed between diapausing and non-diapausing females (Figure 1, Table S1). Lirakis et al. [37] suggested redefining diapause as a stress response to cold temperatures. They suggested that diapause-scoring methods, like the one applied here, may underestimate the true degree of diapause. In fact, they showed that oogenesis was blocked at either a previtellogenic or a very early vitellogenic stage when flies were exposed to dormancy-inducing conditions. This might be the reason underlying the relatively modest number of DEGs identified here, as compared with the photoperiodic experiment. Only a few biological functions were enriched in our diapause DEGs, with some genes being clearly related to diapause (e.g., *yp2-3*, *Egfr*; Table S1). In the photoperiodic experiment, the number of DEGs was substantially higher (517; Figure 1, Table S1). Over-represented functions in this list included “oxidoreduction”, “acyl-CoA metabolism”, and, in particular, “alternative splicing”. “Alternative splicing” and “splice variants” were also enriched functions among the long-day AGO-1 immunoprecipitates transcripts. Alternative splicing of circadian clock genes has been previously implicated in the response to seasonal changes, expressed by photoperiods or temperatures [38,39,40,41]. Our results expand these data, suggesting that alternative splicing may confer a plastic response to environmental changes in *Drosophila*.

Whether the circadian clock is required for photoperiodic timing in flies is not yet clear, although, recent evidence suggests a photoperiodic role for clock PDF-expressing neurons [5]. Our list of photoperiod DEGs included *vri* and *Shaggy* (*Sgg*) that may serve as a link between the photoperiod and circadian clocks [22,23,42,43,44,45].

Two clock genes (*Clk* and *vri*) were also among the miRNA targets (long-day) that we identified by the AGO-1 immunoprecipitation. In a screen for diapause genes in *D. montana*, *cpo* and the circadian clock genes *vri* and *per* were seen to be differentially expressed [46]. *per* and *cpo* were associated with the diapause initiation phase, while *vri* was associated with the diapause maintenance phase. In agreement with Salminen et al. [46], our photoperiodic DEGs included genes encoding the *myosin-heavy chain* (*Mhc*), *pyrroline 5-carboxylate reductase* (*P5cr2*), and *Hsp70s*. 

Interestingly, our photoperiodic DEGs also included a number of odorant-binding protein genes (i.e., *Obp56g*, *Obp99a*, *Obp99b*, *Obp83g*, and *Obp49a*). Similar genes have been previously associated with diapause in *D. montana* [47]. *Obp99b* is repressed by JH, which is produced by the *corpora allata* [24] and was found to be upregulated in diapause [6]. There is a growing recognition that *Obps* are involved in multiple processes, such as desiccation resistance, immune system development, and aggression [48]. Correspondingly, the expression pattern of *Obp* is diverse and not limited to the olfactory sensilla on the antenna. Natural polymorphism in *Obp99b* is associated with longevity [49], and increased lifespan was observed in odorant receptor mutant *Orb83b* [50]. Intriguingly, the increased longevity in *Orb83b* mutant is not due to insulin signaling, which is generally considered a major agent that interlink aging and diapause [50]. 

The network analysis presented here revealed additional pathways that are associated with both diapause and the photoperiodic response. A unique function that was enriched among diapause DEGs was “fat body development”. Indeed, the fat bodies are responsible for the production of yolk proteins (YP) that are fundamental components of the developing oocyte. During diapause, the production of *insulin-like peptides* (*ILPs*) is repressed, resulting in a repression of *JH* and *ecdison* expression, which in turn inhibits the synthesis of YP in the fat bodies [3]. Other pathways that were shared between the miRNA and photoperiodic networks fit very well with the expected diapause–hormonal axis. These pathways include “ecdysone receptor-mediated signaling”, “positive regulation of hormone secretion”, and “phospholipid biosynthetic process”. The pathway “rhodopsin mediated signaling” was shared by both the diapause and miRNA networks, and may constitute a possible link between light perception (compound eyes, ocelli) and the IPCs responsible for ILPs production. *EcR* and other genes of the insulin pathway (i.e., *InR*, *Ilp6*; Table S6) were indeed enriched in long-day flies in our AGO immunoprecipitation experiments. The photoperiodic network included unique biological processes, such as “rRNA methylation”, “histone phosphorylation”, “regulation of circadian sleep/wake”, and “gene silencing by miRNA” (Table S5). The other functions associated with this network underscore the significance of mechanisms that regulate transcription (e.g., alternative splicing histone modifications, miRNA-dependent silencing) to the photoperiodic response. Functions shared by the diapause and the photoperiodic networks (i.e., “tRNA methylation” and “regulation of translational initiation”) reinforce this notion. Interestingly, biological functions shared by both the photoperiodic and miRNA networks (i.e., “entrainment of circadian clock”, “regulation of circadian rhythm”, “response to heat”, and “visual behavior”) (Table S5) were also associated with the diapause response in *D. virillis* [46]. Intersecting all the networks produced two interaction hubs of genes (Figure 3), represented in a number of important functions, including “fatty acid biosynthetic process”, “positive regulation of hormone secretion”, “regulation of transcription”, “regulation of circadian sleep/wake cycle”, “positive regulation of apoptosis”, “ecdysone receptor-mediated signaling”, “determination of adult lifespan”, “nuclear mRNA splicing”, “olfactory behavior”, and “Notch signaling”. Finally, biological functions, such as “acid biosynthetic”, “acyl-CoA metabolism”, and “oxido-reduction”, that we identified here, were associated with diapause in previous studies in other *Drosophila* species [46,47]. 

## 4. Materials and Methods

### 4.1. Flies Maintenance and Samples Collection

Canton-S flies were maintained in 200 mL glass bottles containing fly food (4.6% sugar, 4.6% brewer’s yeast, 1.25% agar, 0.2% methyl 4-hydroxybenzoate) at 25 °C in a 12 h light–12 h dark cycle (LD 12:12). Flies were collected in a 6 h post-eclosion window and maintained at 12.5 ± 0.2 °C for long (LD 16:8) and short (LD 8:16) photoperiods (for temperature trace, see Figure S2), hereafter referred to as long-day photoperiod and short-day photoperiod, respectively. The flies were maintained in 2 × 10 cm plastic vials in 12.5 × 19 × 26 cm chambers illuminated by a white fluorescent tube (standard T5 F4W/33). Twelve days later, female heads were collected in dry ice 4–6 h after lights-on and stored at −80 °C in TRIzol reagent (Invitrogen). Each sample was tested for the level of diapause based on previously described stages of ovary development [51]. A fly was considered to be in reproductive arrest if its most advanced oocyte was previtellogenic (i.e., prior to stage 8). For overexpressing miRNA in *Pdf* neurons, we crossed UAS-miRNA flies (Bloomington stock number: 41128, 41172, 41174, 41166) with PDF-Gal4 flies (Bloomington stock number: 6900). As control experiments, both the UAS-miRNAs and PDF-Gal4 strains were crossed with flies presenting a *yellow-white* genetic background. The levels of diapause were recorded in these samples as described above.

### 4.2. MiRNA and Gene Expression Profiling

Total RNA was isolated using TRIzol reagent (Invitrogen). The miRNA fraction was isolated from each sample using the pureLink miRNA isolation kit (Invitrogen). Subsequently, the NCode multispecies miRNA microarray v2 (Invitrogen) was hybridized with fluorescently labeled miRNAs from flies experiencing both long and short days. The NCode multispecies miRNA microarray v2 alone was used as a positive control. The experiment was repeated 4 times. After hybridization, chip images were scanned using GenePix 4.1 software. Signal intensity was analyzed using the Limma package of R software [52]. Possible target genes for differentially expressed miRNAs were identified using Target scan [53] and Pictar [54]. DAVID software [55] was used to search for enriched biological functions amongst the identified targets. 

Network analysis was carried out using Cytoscape [25]. We reduced the initial complexity of the network by considering only the most significant interactions. With the Finley dataset, we included the interactions that were verified in a matrix screen, with verified IST and with reporter activity higher than 3 in a 0–8 scale. The Curagen dataset included a confidence score generated using a statistical model that determined attributes that correlate with the likelihood of being in the true or false positive training set. The threshold between high and low confidence interactions was set to 0.5, and only interactions of 0.8 or higher value were included. Interactions from the Hybrigenis dataset were only those that had verified IST and with ISTS RFCS equal or higher than 2 w. See DroID web page (http://www.droidb.org/DBdescription.jsp (accessed on 25 April 2022)). 

For gene expression profiling, RNA was extracted from female heads following 12 days at 12 ± 0.2 °C. In the photoperiodic experiment, females were maintained at LD (16:8) and SD (8:16) conditions. In the diapause experiment, the females were maintained at intermediate LD (14:10) that is expected to drive diapause in 50% of the females (i.e., critical daylength). the antisense copy RNA (cRNA) samples (4 replicates of each condition) were hybridized with GeneChip^®^ Drosophila Genome2.0Array, and data were analyzed using the Limma package in R/Bioconductor. 

### 4.3. AGO-1 Immunoprecipitation and RNA Sequencing

AGO-1 immunoprecipitation was carried out using a previously described protocol [56] with minor modifications. A measure of 200 μL of frozen fly heads where homogenized in 600 μL of lysis buffer (30 mM HEPES KOH pH 7.4, 100 mM KAcetate, 2 mM MgAcetate, 5 percent glycerol, 0.1 percent Triton X-100, 1 mM EGTA, 5 mM DTT, 0.4 U/ul RNAse OUT [Invitrogen], 25 mM EDTA). The lysate was incubated in ice for 10 min and centrifuged at 14,000× *g* rpm for 15 min at 4 °C. A measure of 100 μL of the supernatant was isolated as input while the rest (450 μL) was used for the immunoprecipitation. A measure of 15 μL of anti-AGO-1 antibody was added to the supernatant and incubated at 4 °C overnight with rotation. A measure of 100 μL of protein-G plus beads slurry (MERCK, IP04-1.5ML) was washed trice with 250 μL of lysis buffer and then added to the lysate and incubated at 4 °C for 4 h. The beads were recovered by a 2 min centrifugation at 8000× *g* rcf and washed 5 times in 500 μL lysis buffer at 4 °C with rotation. Finally, the RNA in the immunoprecipitation and input was isolated with TRIzol (Thermo-Fisher Scientific, Waltham, MA, USA) following manufacturer’s instructions. 

RNA-seq library preparation and sequencing were carried out by Glasgow Polyomics (www.polyomics.gla.ac.uk (accessed on 25 April 2022)) using the IlluminaNextseq500 platform. Two independent libraries (pair-end) for long-day and one for short-day (input and IP) flies were generated. Adapter sequences and poor-quality readings were trimmed from the RNA-seq data, and the quality of the resulting trimmed libraries was checked with fastQC (0.11.2) [57]. Between 14.1 and 16.8 Mbp of sequence per library with “per base quality score” > 30 phred and “mean per sequence quality score” > 33 phred were obtained. The sequences were aligned to the *D. melanogaster* transcriptome downloaded from the illumina igenome website. For the alignment we used the software STAR (version 2.5.2b). Samtools (version 1.3.2) [58] was used to convert sam to bam files to sort them and index them. To quantify the expression of transcripts (genes) of the aligned RNA, we initially used cufflinks (version 2.2.1), cuffmerge, and cuffquant [59]. The expression was expressed in fragments per kilobase of transcript per million fragments mapped (FPKM). Finally, to calculate enriched transcripts in IP (input vs. IP) and differences between samples (long vs. short day), we used cuffdiff, with a geometric method of normalization [60]. This process generated lists of enriched transcripts (FDR q < 0.05) in long and short days as well as a list of differentially enriched transcripts (long vs. short day). 

## Data Availability

Gene expression and RNA sequencing data can be accessed at Gene Expression Omnibus (GEO, https://www.ncbi.nlm.nih.gov/geo/ (accessed on 25 April 2022)) accession number: GSE28588, GSE28370, GSE28246, and GSE189992.

## Supplementary Materials

The following supporting information can be downloaded at: https://www.mdpi.com/article/10.3390/ijms23094935/s1.
