# Supplementary material for: Photoperiod-Dependent Expression of MicroRNA in Drosophila
Source: Int J Mol Sci. 2022 Apr 29;23(9):4935. doi: 10.3390/ijms23094935 (PMC9100521; doi:10.3390/ijms23094935)
Supplement: Supplementary file 1 [file ijms-23-04935-s001.zip › ijms-1553125-supplementary.pdf]

# Supplemental Information

## Photoperiod-dependent expression of MicroRNA in *Drosophila*

**Mirko Pegoraro [1], Bettina Fishman [2], Valeria Zonato [3], Georgios Zouganelis [4], Amanda Francis [1], Charalambos P. Kyriacou [3] and Eran Tauber [2,*]**

## Content

**Table S1.** Diapause and Photoperiodic DEGs

**Table S2.** GO term analysis

**Table S3**. Diapause and Photoperiodic common DEGs

**Table S4**. Top 20 predicted targets of DEMs

**Table S5**. Networks-associated GO Biological functions

**Table S6**. AGO-1 Immunoprecipitation Enriched transcripts

**Table S7**. Long day and short day immunoprecipitation common enriched genes

**Figure S1**. Enriched biological functions among AGO-1 immunoprecipitation transcripts

**Figure. S2.** Temperature measurements in the light boxes

## Table S1 Diapause and Photoperiodic DEGs

| Diapause DEGs | | | | | | | |
|---|---|---|---|---|---|---|---|
| **FlyBase ID** | **Gene** | **FlyBase ID** | **Gene** | **FlyBase ID** | **Gene** | **FlyBase ID** | **Gene** |
| *FBgn0033170* | *sPLA2* | *FBgn0026873* | *MED18* | *FBgn0053080* | *CG33080* | *FBgn0029959* | *Rab39* |
| *FBgn0038795* | *CG4335* | *FBgn0000477* | *DNaseII* | *FBgn0037012* | *Rcd2* | *FBgn0027538* | *beta4GalNAcTA* |
| *FBgn0026428* | *HDAC6* | *FBgn0035484* | *CG11594* | *FBgn0039611* | *CG14528* | *FBgn0038034* | *Cyp9f3Psi* |
| *FBgn0003731* | *Egfr* | *FBgn0032820* | *fbp* | *FBgn0029765* | *CG16756* | *FBgn0034117* | *CG7997* |
| *FBgn0035372* | *CG12093* | *FBgn0051778* | *CG31778* | *FBgn0005391* | *Yp2* | *FBgn0036695* | *Papst2* |
| *FBgn0025469* | *slv* | *FBgn0034646* | *Rae1* | *FBgn0030351* | *CG1840* | *FBgn0034688* | *CG11474* |
| *FBgn0033820* | *CG4716* | *FBgn0039023* | *CG4723* | *FBgn0034010* | *CG8157* | *FBgn0030582* | *CG14411* |
| *FBgn0053120* | *CG33120* | *FBgn0035667* | *Jon65Ai* | *FBgn0053493* | *CG33493* | *FBgn0004797* | *mdy* |
| *FBgn0028955* | *CG8788* | *FBgn0027594* | *drpr* | *FBgn0034181* | *CG8963* | *FBgn0004554* | *Edg91* |
| *FBgn0014000* | *Hf* | *FBgn0260632* | *dl* | *FBgn0034491* | *Hsl* | *FBgn0061356* | *CG18003* |
| *FBgn0037885* | *CG17721* | *FBgn0030160* | *CG9691* | *FBgn0043783* | *CG32444* | *FBgn0263131* | *CG43373* |
| *FBgn0038463* | *CG3534* | *FBgn0033859* | *fand* | *FBgn0259832* | *CG34229* | *FBgn0260388* | *CG42514* |
| *FBgn0034913* | *Snap29* | *FBgn0035091* | *CG3829* | *FBgn0015336* | *CG15865* | *FBgn0263131* | *CG43373* |
| *FBgn0004888* | *Scsalpha* | *FBgn0028519* | *hll* | *FBgn0040606* | *CG6503* | *FBgn0260632* | *dl* |
| *FBgn0024314* | *Plap* | *FBgn0011705* | *rost* | *FBgn0025592* | *Gk1* | *FBgn0020389* | *Papss* |
| *FBgn0034887* | *St1* | *FBgn0054054* | *CG34054* | *FBgn0031360* | *CG31937* | *FBgn0050083* | *CG30083* |
| *FBgn0039031* | *Gbp3* | *FBgn0015025* | *CkIIalpha-i1* | *FBgn0052163* | *CG32163* | *FBgn0031950* | *Herp* |
| *FBgn0034110* | *Atg9* | *FBgn0031139* | *CG11227* | *FBgn0039827* | *CG1544* | *FBgn0051217* | *modSP* |
| *FBgn0004177* | *mts* | *FBgn0033232* | *CG12159* | *FBgn0035734* | *CG14823* | *FBgn0028683* | *spt4* |
| *FBgn0032218* | *CG5381* | *FBgn0032940* | *Mondo* | | | | |
| *FBgn0033377* | *Pmm45A* | *FBgn0039150* | *CG13605* | | | | |
| *FBgn0051445* | *CG31445* | *FBgn0000592* | *Est-6* | | | | |
| *FBgn0085424* | *nub* | *FBgn0038313* | *CG4338* | | | | |

| | | | |
|---|---|---|---|
| *FBgn0023545* | *CG4406* | *FBgn0028526* | *CG15293* |
| *FBgn0031022* | *CG12204* | *FBgn0033188* | *Drat* |
| *FBgn0039678* | *Obp99a* | *FBgn0011204* | *cue* |
| *FBgn0040089* | *meso18E* | *FBgn0035107* | *mri* |
| *FBgn0025366* | *Ip259* | *FBgn0261445* | *sgl* |
| *FBgn0033799* | *GLaz* | *FBgn0002719* | *Men* |
| *FBgn0035090* | *CG2736* | *FBgn0034786* | *CG13531* |
| *FBgn0087007* | *bbg* | *FBgn0036549* | *CG10516* |
| *FBgn0259977* | *Tdc1* | *FBgn0033564* | *Pex6* |
| *FBgn0036208* | *CG10361* | *FBgn0037230* | *CG9780* |
| *FBgn0036157* | *CG7560* | *FBgn0004047* | *Yp3* |
| *FBgn0024321* | *NK7.1* | *FBgn0027930* | *MP1* |
| *FBgn0050015* | *CG30015* | *FBgn0027506* | *EDTP* |
| *FBgn0000635* | *Fas2* | *FBgn0052667* | *ssp7* |
| *FBgn0261552* | *ps* | *FBgn0033086* | *CG9410* |
| *FBgn0038966* | *pinta* | *FBgn0265177* | *CG44242* |
| *FBgn0040234* | *c12.2* | *FBgn0265178* | *CG44243* |
| *FBgn0265297* | *pAbp* | *FBgn0027579* | *mino* |
| *FBgn0029079* | *icln* | *FBgn0013435* | *Cdc2rk* |
| *FBgn0033373* | *CG8080* | *FBgn0034390* | *CG15093* |
| *FBgn0261279* | *lqfR* | *FBgn0031417* | *CG3597* |
| *FBgn0039022* | *CG4725* | *FBgn0050090* | *CG30090* |
| *FBgn0039766* | *CG15536* | *FBgn0031498* | *CG17260* |
| *FBgn0050148* | *CG30148* | *FBgn0037222* | *CG14642* |
| *FBgn0027610* | *Dic1* | *FBgn0051719* | *RluA-1* |
| *FBgn0000316* | *cin* | *FBgn0029769* | *frma* |
| *FBgn0002565* | *Lsp2* | *FBgn0004370* | *Ptp10D* |
| *FBgn0036732* | *Oatp74D* | *FBgn0035232* | *CG12099* |
| *FBgn0035449* | *CG14971* | *FBgn0037087* | *CG7519* |
| *FBgn0027550* | *CG6495* | *FBgn0259175* | *ome* |

| Photoperiod DEGs | | | | | | | |
|---|---|---|---|---|---|---|---|
| **FlyBase ID** | **Gene** | **FlyBase ID** | **Gene** | **FlyBase ID** | **Gene** | **FlyBase ID** | **Gene** |
| *FBgn0003380* | *Sh* | *FBgn0264270* | *Sxl* | *FBgn0261239* | *Hr39* | *FBgn0025595* | *AkhR* |
| *FBgn0015037* | *Cyp4p1* | *FBgn0037556* | *CG9636* | *FBgn0264495* | *gpp* | *FBgn0267001* | *Ten-a* |
| *FBgn0033055* | *Tbce* | *FBgn0024806* | *DIP2* | *FBgn0035911* | *CG6638* | *FBgn0015039* | *Cyp9b2* |
| *FBgn0262714* | *Sap130* | *FBgn0038473* | *Ns1* | *FBgn0039955* | *CG41099* | *FBgn0040297* | *Nhe2* |
| *FBgn0039789* | *CG9717* | *FBgn0027341* | *Gfat1* | *FBgn0032731* | *Swip-1* | *FBgn0038601* | *CG18600* |
| *FBgn0031695* | *Cyp4ac3* | *FBgn0000032* | *Acph-1* | *FBgn0039800* | *Npc2g* | *FBgn0264270* | *Sxl* |
| *FBgn0033465* | *Etf-QO* | *FBgn0038142* | *CheA87a* | *FBgn0036366* | *CG10133* | *FBgn0026593* | *CG5707* |
| *FBgn0026077* | *Gasp* | *FBgn0050296* | *RIC-3* | *FBgn0039830* | *ATPsynC* | *FBgn0032388* | *CG6686* |
| *FBgn0261283* | *SREBP* | *FBgn0032957* | *CG2225* | *FBgn0039114* | *Lsd-1* | *FBgn0020513* | *ade5* |
| *FBgn0031692* | *TpnC25D* | *FBgn0039316* | *CG11893* | *FBgn0038388* | *CG4287* | *FBgn0039767* | *CG2218* |
| *FBgn0032690* | *CG10333* | *FBgn0040238* | *Best1* | *FBgn0036460* | *CG5114* | *FBgn0027111* | *miple1* |
| *FBgn0020503* | *CLIP-190* | *FBgn0033543* | *CG12338* | *FBgn0001092* | *Gapdh2* | *FBgn0052767* | *CG32767* |
| *FBgn0035141* | *Cypl* | *FBgn0032699* | *CG10383* | *FBgn0020372* | *TM4SF* | *FBgn0036365* | *cmb* |
| *FBgn0029996* | *UbcE2H* | *FBgn0050148* | *CG30148* | *FBgn0051174* | *CG31174* | *FBgn0033101* | *CG9436* |
| *FBgn0053120* | *CG33120* | *FBgn0265180* | *CG44245* | *FBgn0011206* | *bol* | *FBgn0015569* | *alpha-Est10* |
| *FBgn0035791* | *CG8539* | *FBgn0265191* | *Glycogenin* | *FBgn0050017* | *CG30017* | *FBgn0032167* | *CG5853* |
| *FBgn0024989* | *CG3777* | *FBgn0041181* | *Tep3* | *FBgn0027779* | *VhaSFD* | *FBgn0267252* | *Ggamma30A* |
| *FBgn0024182* | *waw* | *FBgn0046114* | *Gclm* | *FBgn0051475* | *CG31475* | *FBgn0053494* | *CG33494* |
| *FBgn0261985* | *Ptpmeg* | *FBgn0016076* | *vri* | *FBgn0004373* | *fwd* | *FBgn0017579* | *RpL14* |
| *FBgn0024189* | *sns* | *FBgn0031459* | *CG2862* | *FBgn0086778* | *nAChRalpha7* | *FBgn0050345* | *CG30345* |
| *FBgn0033065* | *Cyp6w1* | *FBgn0002565* | *Lsp2* | *FBgn0032297* | *CG17124* | *FBgn0266284* | *Ns3* |
| *FBgn0014000* | *Hf* | *FBgn0032979* | *Clamp* | *FBgn0283521* | *lola* | *FBgn0014031* | *Spat* |
| *FBgn0035164* | *CG13901* | *FBgn0038834* | *RpS30* | *FBgn0260441* | *RpS12* | *FBgn0053170* | *CG33170* |
| *FBgn0034141* | *CG8311* | *FBgn0004118* | *nAChRbeta2* | *FBgn0083978* | *CG17672* | *FBgn0029799* | *CG15772* |
| *FBgn0022288* | *l(2)09851* | *FBgn0085201* | *CG34172* | *FBgn0021765* | *scu* | *FBgn0261260* | *mgl* |
| *FBgn0052676* | *stx* | *FBgn0032008* | *CG14277* | *FBgn0040392* | *CG14050* | *FBgn0038721* | *subdued* |
| *FBgn0028540* | *CG9008* | *FBgn0028743* | *Dhit* | *FBgn0016701* | *Rab4* | *FBgn0004045* | *Yp1* |
| *FBgn0052425* | *CG32425* | *FBgn0003512* | *Sry-delta* | *FBgn0027086* | *IleRS* | *FBgn0259707* | *CG42361* |
| *FBgn0036659* | *CG9701* | *FBgn0085442* | *NKAIN* | *FBgn0037981* | *Spt3* | *FBgn0000504* | *dsx* |

| | | | | | | | |
|---|---|---|---|---|---|---|---|
| *FBgn0267823* | *Gmer* | *FBgn0005391* | *Yp2* | *FBgn0040064* | *yip2* | *FBgn0031474* | *CG2991* |
| *FBgn0037553* | *CG18249* | *FBgn0033015* | *d4* | *FBgn0039525* | *CG5646* | *FBgn0263740* | *eIF-2gamma* |
| *FBgn0039406* | *RpL34a* | *FBgn0003360* | *sesB* | *FBgn0039543* | *CG12428* | *FBgn0263755* | *Su(var)3-9* |
| *FBgn0033665* | *Zip48C* | *FBgn0266579* | *tau* | *FBgn0032394* | *CG6746* | *FBgn0038398* | *sxe2* |
| *FBgn0260441* | *RpS12* | *FBgn0038092* | *beat-Vb* | *FBgn0023542* | *Nmd3* | *FBgn0038984* | *AdipoR* |
| *FBgn0030529* | *Clic* | *FBgn0036389* | *ssp2* | *FBgn0037705* | *mura* | *FBgn0027279* | *l(1)G0196* |
| *FBgn0001228* | *CG4456* | *FBgn0045064* | *bwa* | *FBgn0026565* | *CG1315* | *FBgn0283536* | *Vha13* |
| *FBgn0261625* | *GLS* | *FBgn0032433* | *Oatp33Ea* | *FBgn0013763* | *Idgf6* | *FBgn0039932* | *fuss* |
| *FBgn0261673* | *nemy* | *FBgn0029761* | *SK* | *FBgn0034808* | *CG9896* | *FBgn0265998* | *Doa* |
| *FBgn0003062* | *Fib* | *FBgn0259682* | *Jabba* | *FBgn0010052* | *Jhe* | *FBgn0051998* | *CG31998* |
| *FBgn0032135* | *GlcAT-S* | *FBgn0085443* | *spri* | *FBgn0045761* | *CHKov1* | *FBgn0000477* | *DNaseII* |
| *FBgn0033699* | *RpS11* | *FBgn0037702* | *CG8176* | *FBgn0034420* | *CG10737* | *FBgn0016926* | *Pino* |
| *FBgn0029170* | *TwdlT* | *FBgn0040334* | *Tsp3A* | *FBgn0035756* | *unc-13-4A* | *FBgn0265003* | *koi* |
| *FBgn0003079* | *Raf* | *FBgn0261955* | *kdn* | *FBgn0034474* | *Obp56g* | *FBgn0035186* | *CG13912* |
| *FBgn0086674* | *Tango13* | *FBgn0263929* | *jvl* | *FBgn0024352* | *Hop* | *FBgn0262109* | *CR42862* |
| *FBgn0024734* | *PRL-1* | *FBgn0033782* | *sug* | *FBgn0032414* | *CG17211* | *FBgn0034094* | *Tsf3* |
| *FBgn0029896* | *CG3168* | *FBgn0039008* | *CG6972* | *FBgn0085399* | *CG34370* | *FBgn0086450* | *su(r)* |
| *FBgn0035346* | *CG1146* | *FBgn0034918* | *Pym* | *FBgn0039678* | *Obp99a* | *FBgn0051778* | *CG31778* |
| *FBgn0031939* | *CG13796* | *FBgn0039024* | *CG4721* | *FBgn0035632* | *Ppat-Dpck* | *FBgn0003371* | *sgg* |
| *FBgn0037842* | *CG6567* | *FBgn0037207* | *Mes2* | *FBgn0031945* | *CG7191* | *FBgn0035495* | *CG14989* |
| *FBgn0013269* | *FK506-bp1* | *FBgn0003209* | *raw* | *FBgn0024293* | *Spn43Ab* | *FBgn0003721* | *Tm1* |
| *FBgn0262167* | *ana1* | *FBgn0039897* | *CG1674* | *FBgn0038914* | *fit* | *FBgn0039161* | *CG13606* |
| *FBgn0052311* | *zormin* | *FBgn0259716* | *CG42370* | *FBgn0036157* | *CG7560* | *FBgn0264001* | *bru3* |

**Photoperiod DEGs**

| FlyBase ID | Gene | FlyBase ID | Gene | FlyBase ID | Gene | FlyBase ID | Gene |
|---|---|---|---|---|---|---|---|
| *FBgn0034497* | *CG9090* | *FBgn0001122* | *Galphao* | *FBgn0030575* | *CG5321* | *FBgn0022984* | *qkr58E-3* |
| *FBgn0010488* | *NAT1* | *FBgn0261283* | *SREBP* | *FBgn0086679* | *p* | *FBgn0027930* | *MP1* |
| *FBgn0032472* | *CG9928* | *FBgn0004698* | *Xpc* | *FBgn0041775* | *tral* | *FBgn0028703* | *Nhe3* |
| *FBgn0266848* | *wap* | *FBgn0270926* | *AsnS* | *FBgn0023516* | *Pex5* | *FBgn0034804* | *CG3831* |
| *FBgn0029666* | *CG10803* | *FBgn0032499* | *Uvrag* | *FBgn0031801* | *CG9498* | *FBgn0038300* | *Mau2* |

| | | | | | | | |
|---|---|---|---|---|---|---|---|
| *FBgn0039667* | *CG2010* | *FBgn0010516* | *wal* | *FBgn0264975* | *Nrg* | *FBgn0042135* | *CG18812* |
| *FBgn0031959* | *spz3* | *FBgn0031390* | *tho2* | *FBgn0004657* | *mys* | *FBgn0033807* | *AQP* |
| *FBgn0031227* | *CG3709* | *FBgn0261808* | *cu* | *FBgn0020909* | *Rtc1* | *FBgn0051871* | *CG31871* |
| *FBgn0032029* | *CG17292* | *FBgn0052137* | *CG32137* | *FBgn0015279* | *Pi3K92E* | *FBgn0010575* | *sbb* |
| *FBgn0004587* | *B52* | *FBgn0013279* | *Hsp70Bc* | *FBgn0034247* | *CG6484* | *FBgn0010434* | *cora* |
| *FBgn0051361* | *dpr17* | *FBgn0053519* | *Unc-89* | *FBgn0024555* | *flfl* | *FBgn0010100* | *Acon* |
| *FBgn0035688* | *CG10289* | *FBgn0003067* | *Pepck* | *FBgn0002772* | *Mlc1* | *FBgn0036302* | *sowah* |
| *FBgn0004654* | *Pgd* | *FBgn0017482* | *T3dh* | *FBgn0032925* | *CG9246* | *FBgn0022160* | *Gpo-1* |
| *FBgn0022787* | *Hel89B* | *FBgn0026576* | *CG5991* | *FBgn0025726* | *unc-13* | *FBgn0035521* | *VhaM9.7-a* |
| *FBgn0265045* | *Strn-Mlck* | *FBgn0010482* | *l(2)01289* | *FBgn0050273* | *CG30273* | *FBgn0028336* | *l(1)G0255* |
| *FBgn0036549* | *CG10516* | *FBgn0035999* | *CG3552* | *FBgn0013765* | *cnn* | *FBgn0264491* | *how* |
| *FBgn0035452* | *CG10359* | *FBgn0035817* | *CG7409* | *FBgn0022023* | *eIF-3p40* | *FBgn0038975* | *Nrx-1* |
| *FBgn0039626* | *Slu7* | *FBgn0013276* | *Hsp70Ab* | *FBgn0027334* | *l(1)G0004* | *FBgn0040777* | *CG14767* |
| *FBgn0031813* | *CG9527* | *FBgn0035515* | *CG14997* | *FBgn0001223* | *Hsp22* | *FBgn0032363* | *Dlg5* |
| *FBgn0004047* | *Yp3* | *FBgn0031857* | *CG11321* | *FBgn0039136* | *CG5902* | *FBgn0030178* | *CG2974* |
| *FBgn0032407* | *Pex19* | *FBgn0032299* | *CG17127* | *FBgn0015570* | *alpha-Est2* | *FBgn0037370* | *CG1236* |
| *FBgn0038115* | *CG7966* | *FBgn0037636* | *CG9821* | *FBgn0031146* | *CG15449* | *FBgn0025936* | *Eph* |
| *FBgn0043792* | *CG30427* | *FBgn0020305* | *dbe* | *FBgn0267376* | *SelR* | *FBgn0263830* | *CG40486* |
| *FBgn0052095* | *CG32095* | *FBgn0011642* | *Zyx* | *FBgn0036053* | *iPLA2-VIA* | *FBgn0037491* | *CG1227* |
| *FBgn0265991* | *Zasp52* | *FBgn0011288* | *Snap25* | *FBgn0000244* | *by* | *FBgn0032151* | *nAChRalpha6* |
| *FBgn0013277* | *Hsp70Ba* | *FBgn0261673* | *nemy* | *FBgn0033226* | *CG1882* | *FBgn0031940* | *CG7214* |
| *FBgn0283471* | *wupA* | *FBgn0034162* | *CG6426* | *FBgn0036824* | *CG3902* | *FBgn0040322* | *GNBP2* |
| *FBgn0032430* | *CG6388* | *FBgn0019985* | *mGluR* | *FBgn0030723* | *dpr18* | *FBgn0085783* | *CR41111* |
| *FBgn0004117* | *Tm2* | *FBgn0030731* | *Mfe2* | *FBgn0038964* | *Nop56* | *FBgn0013278* | *Hsp70Bb* |
| *FBgn0262476* | *CG43066* | *FBgn0022986* | *qkr58E-1* | *FBgn0038395* | *CG10407* | *FBgn0027844* | *CAH1* |
| *FBgn0040020* | *MED21* | *FBgn0037758* | *CG9467* | *FBgn0026147* | *CG16833* | *FBgn0022800* | *Cad96Ca* |
| *FBgn0027580* | *PCB* | *FBgn0034943* | *Fmo-1* | *FBgn0038742* | *Arc42* | *FBgn0003423* | *slgA* |
| *FBgn0037093* | *Cdk12* | *FBgn0037328* | *RpL35A* | *FBgn0024921* | *Trn* | *FBgn0029608* | *CG3091* |
| *FBgn0031417* | *CG3597* | *FBgn0026196* | *nop5* | *FBgn0040208* | *Kat60* | *FBgn0002643* | *mam* |
| *FBgn0083969* | *CG34133* | *FBgn0011768* | *Fdh* | *FBgn0038795* | *CG4335* | *FBgn0010015* | *CanA1* |
| *FBgn0264894* | *CG44085* | *FBgn0039685* | *Obp99b* | *FBgn0037443* | *Dmtn* | *FBgn0262515* | *VhaAC45* |

| | | | | | | | |
|---|---|---|---|---|---|---|---|
| *FBgn0037525* | *CG17816* | *FBgn0038038* | *CG5167* | *FBgn0052113* | *CG32113* | *FBgn0037819* | *CG14688* |
| *FBgn0264695* | *Mhc* | *FBgn0039226* | *Ude* | *FBgn0039805* | *Cpr100A* | *FBgn0000459* | *disco* |
| *FBgn0050269* | *CG30269* | *FBgn0004465* | *Su(P)* | *FBgn0263197* | *Patronin* | *FBgn0052264* | *CG32264* |
| *FBgn0029914* | *CG4558* | *FBgn0032587* | *CG5953* | *FBgn0000715* | *FMRFa* | *FBgn0017581* | *Lk6* |
| *FBgn0032819* | *CG10463* | *FBgn0051781* | *CR31781* | *FBgn0039266* | *CG11791* | *FBgn0262509* | *nrm* |
| *FBgn0010292* | *bys* | *FBgn0005536* | *Mbs* | *FBgn0034158* | *CG5522* | *FBgn0037090* | *Est-Q* |
| *FBgn0029828* | *CG6067* | *FBgn0030828* | *CG5162* | *FBgn0039151* | *CG13607* | *FBgn0011284* | *RpS4* |
| *FBgn0003862* | *trx* | *FBgn0039620* | *wat* | *FBgn0038552* | *CG18012* | *FBgn0039993* | *CG17691* |
| *FBgn0037439* | *CG10286* | *FBgn0032785* | *CG10026* | *FBgn0029714* | *CG3527* | *FBgn0052016* | *4E-T* |
| *FBgn0032701* | *CG10341* | *FBgn0013275* | *Hsp70Aa* | *FBgn0032881* | *CG9319* | *FBgn0086904* | *Nacalpha* |
| *FBgn0036939* | *CG7365* | *FBgn0261341* | *verm* | *FBgn0004913* | *Gnf1* | *FBgn0001208* | *Hn* |
| *FBgn0004404* | *RpS14b* | *FBgn0034027* | *CG8187* | *FBgn0035162* | *CG13900* | *FBgn0259481* | *Mob2* |
| *FBgn0001187* | *Hex-C* | *FBgn0028479* | *Mtpalpha* | *FBgn0025352* | *Thiolase* | *FBgn0037386* | *CG1208* |
| *FBgn0037408* | *NPFR* | *FBgn0028737* | *Ef1beta* | *FBgn0083963* | *Nlg3* | *FBgn0036377* | *CG10710* |
| *FBgn0038735* | *CG4662* | *FBgn0038516* | *P5cr-2* | *FBgn0039770* | *CG15537* | *FBgn0027590* | *GstE12* |
| *FBgn0034214* | *CG6550* | *FBgn0262593* | *Shab* | *FBgn0259678* | *sqa* | *FBgn0265137* | *Spn42Da* |

| **Photoperiod DEGs** | | | |
|---|---|---|---|
| **FlyBase ID** | **Gene** | **FlyBase ID** | **Gene** |
| *FBgn0038407* | *CG6126* | *FBgn0259937* | *Nop60B* |
| *FBgn0030485* | *CG1998* | *FBgn0027507* | *CG1344* |
| *FBgn0011656* | *Mef2* | *FBgn0026415* | *Idgf4* |
| *FBgn0034440* | *CG10073* | *FBgn0031957* | *TwdlE* |
| *FBgn0035811* | *CG12262* | *FBgn0043796* | *CG12219* |
| *FBgn0010225* | *Gel* | *FBgn0265048* | *cv-d* |
| *FBgn0029941* | *CG1677* | *FBgn0261065* | *Cpsf73* |
| *FBgn0263605* | *l(3)72Dn* | *FBgn0001091* | *Gapdh1* |
| *FBgn0035431* | *CG14968* | *FBgn0035588* | *CG10672* |
| *FBgn0036463* | *Reck* | *FBgn0032048* | *Dh31* |
| *FBgn0263006* | *SERCA* | *FBgn0032264* | *Lip4* |
| *FBgn0040551* | *CG11686* | *FBgn0030234* | *CG15211* |

| | | | |
|---|---|---|---|
| *FBgn0034755* | *CG3746* | *FBgn0261341* | *verm* |
| *FBgn0259224* | *CG42324* | *FBgn0015663* | *Dot* |
| *FBgn0030347* | *CG15739* | *FBgn0038034* | *Cyp9f3Psi* |
| *FBgn0263396* | *sqd* | *FBgn0003392* | *shi* |
| *FBgn0037015* | *cmpy* | *FBgn0030251* | *CG2145* |
| *FBgn0264489* | *CG43897* | *FBgn0050431* | *CG30431* |
| *FBgn0046875* | *Obp83g* | *FBgn0003149* | *Prm* |
| *FBgn0032907* | *CG9272* | *FBgn0035844* | *CG13676* |
| *FBgn0037562* | *CG11671* | *FBgn0000152* | *Axs* |
| *FBgn0035091* | *CG3829* | *FBgn0032836* | *CG10680* |
| *FBgn0261065* | *Cpsf73* | *FBgn0265184* | *CG44249* |
| *FBgn0039260* | *Smg6* | *FBgn0265192* | *Snp* |
| *FBgn0260659* | *CG42542* | *FBgn0034071* | *CG8405* |
| *FBgn0040531* | *CG11741* | *FBgn0015808* | *ScpX* |
| *FBgn0027572* | *CG5009* | *FBgn0263997* | *CG43740* |
| *FBgn0039227* | *polybromo* | *FBgn0004364* | *18w* |
| *FBgn0052010* | *CR32010* | *FBgn0051973* | *Cda5* |
| *FBgn0050052* | *Obp49a* | *FBgn0001128* | *Gpdh* |
| *FBgn0017418* | *ari-1* | *FBgn0033729* | *Cpr49Af* |
| *FBgn0086690* | *Plp* | *FBgn0002773* | *Mlc2* |
| *FBgn0051374* | *sals* | *FBgn0026374* | *Rhp* |
| *FBgn0040375* | *CG13760* | *FBgn0031995* | *CG8475* |
| *FBgn0014417* | *CG13397* | *FBgn0030574* | *CG9413* |
| *FBgn0283658* | *muc* | *FBgn0016122* | *Acer* |
| *FBgn0031703* | *CG12512* | *FBgn0004403* | *RpS14a* |
| *FBgn0031092* | *CG9577* | *FBgn0034075* | *Asph* |
| *FBgn0030321* | *CG1703* | *FBgn0033317* | *CG8635* |
| *FBgn0038105* | *yellow-f2* | *FBgn0017590* | *klg* |
| *FBgn0038053* | *CG18549* | *FBgn0262614* | *pyd* |
| *FBgn0052944* | *CG32944* | *FBgn0036035* | *CG18178* |
| *FBgn0033379* | *Mys45A* | *FBgn0031969* | *pes* |

| | | | |
|---|---|---|---|
| *FBgn0024251* | *bbx* | *FBgn0031952* | *cdc14* |
| *FBgn0035538* | *DopEcR* | *FBgn0037592* | *CG11737* |
| *FBgn0023507* | *D2hgdh* | | |
| *FBgn0010383* | *Cyp18a1* | | |
| *FBgn0002521* | *pho* | | |
| *FBgn0031907* | *CG5171* | | |
| *FBgn0261239* | *Hr39* | | |
| *FBgn0005666* | *bt* | | |
| *FBgn0027532* | *CG7139* | | |

**Table S2. GO term analysis.** Enriched GO terms (DAVID, Benjamini-Hochberg q<0.2) of the DEGs in the photoperiodic and diapause experiments.

**Photoperiod**

**Upregulated**

GO:0000062~fatty-acyl-CoA binding

GO:0033539~fatty acid beta-oxidation using acyl-CoA dehydrogenase

GO:0050660~flavin adenine dinucleotide binding

GO:0055114~oxidation-reduction process

**Downregulated**

GO:0000062~fatty-acyl-CoA binding

GO:0003995~acyl-CoA dehydrogenase activity

GO:0005730~nucleolus

GO:0005739~mitochondrion

GO:0005777~peroxisome

GO:0005811~lipid particle

GO:0006635~fatty acid beta-oxidation

GO:0009055~electron carrier activity

GO:0009267~cellular response to starvation

GO:0016491~oxidoreductase activity

GO:0032040~small-subunit processome

GO:0033539~fatty acid beta-oxidation using acyl-CoA dehydrogenase

GO:0040018~positive regulation of multicellular organism growth

GO:0042254~ribosome biogenesis

GO:0042273~ribosomal large subunit biogenesis

GO:0050660~flavin adenine dinucleotide binding

GO:0052689~carboxylic ester hydrolase activity

GO:0052890~oxidoreductase activity, acting on the CH-CH group of donors, with a flavin as acceptor

GO:0055088~lipid homeostasis

GO:0055114~oxidation-reduction process

**Diapause**

**Upregulated**

GO:0006508~proteolysis

**Downregulated**

GO:0005515~protein binding

GO:0005737~cytoplasm

GO:0005829~cytosol

GO:0005856~cytoskeleton

GO:0005886~plasma membrane

GO:0016328~lateral plasma membrane

GO:0030036~actin cytoskeleton organization

GO:0033181~plasma membrane proton-transporting V-type ATPase complex

GO:0042048~olfactory behavior

GO:0045169~fusome

GO:0048477~oogenesis

**Table S3. Diapause and Photoperiodic common DEGs**.

| **Genes** | **Biological Function** |
|---|---|
| *Yolk protein 2* | vitellogenesis; oogenesis; sex differentiation |
| *CG10516* | unknown |
| *Melanization Protease 1* | melanization defense response |
| *CG3597* | oxidation reduction |
| *Yolk protein 3* | embryonic development |
| *Odorant-binding protein 99a* | response to pheromone; sensory perception of chemical stimulus; olfactory behavior |
| *CG7560* | oxidation reduction; methionine metabolic process |
| *CG33120* | unknown |
| *CG30148* | unknown |
| *Helical Factor* | innate immune response |
| *CG31778* | unknown |
| *CG4335* | oxidation reduction; carnitine biosynthetic process |
| *CG3829* | autophagic cell death; salivary gland cell autophagic cell death |
| *CG42351* | unknown |
| *Cyp9f3Ψ* | oxidation reduction |
| *Deoxyribonuclease II* | DNA catabolic process |
| *Larval serum protein 2* | transport |

**Table S4. Top 20 predicted targets of the differentially expressed miRNAs.**

| *dme-mir-2b* | *dme-mir-11* | *dme-mir-34* | *dme-mir-184* | *dme-mir-184** | *dme-mir-274* | *dme-mir-285* |
|---|---|---|---|---|---|---|
| *comm2* | *comm2* | *Swim* | *Mcr* | *CG12347* | *lectin-21Cb* | *CG10055* |
| *cas* | *HLHmdelta* | *CG1544* | *Tsf2* | *CG10795* | *CG34394* | *mlt* |
| *scylla* | *Sik3* | *Eip74EF* | *CG10217* | *loj* | *ex* | *sna* |
| *CG4911* | *Vps15* | *yemalpha* | *pasi2* | *Wnt4* | *Prat* | *TMS1* |
| *CG8963* | *CG8249* | *wb* | *CG15186* | *CG32379* | *wts* | *Hil* |
| *Sik3* | *milton* | *trbl* | *form3* | *CG10195* | *CG32235* | *Vha100-2* |
| *bun* | *CG5608* | *sqz* | *CG1105* | *CG32613* | *CG7927* | *CG1275* |
| *C15* | *noc* | *Rep2* | *sinu* | *Abl* | *Dsp1* | *CG14073* |
| *CCDC53* | *Ih* | *Pcl* | *rn* | *CG12869* | *Lrch* | *Der-2* |
| *CG16885* | *CG17190* | *porin* | *pasi1* | *CG6923* | *CG10324* | *nyo* |
| *CG42675* | *CG10005* | *Glut1* | *CG6583* | *laza* | *Mcr* | *Sap30* |
| *CG6836* | *CG12950* | *prc* | *Nrx* | *Snp* | *Osi7* | *dy* |
| *Vps15* | *CG3534* | *Dok* | *CG10710* | *hbs* | *stau* | *neur* |
| *CG11044* | *EG:22E5.9* | *CG30062* | *Gli* | *CG7888* | *T48* | *vn* |
| *CG13041* | *GLaz* | *smi35A* | *calsyntenin-1* | *Lar* | *hppy* | *if* |
| *ld14* | *gd* | *kkv* | *Sema-1b* | *CG13741* | *Cbl* | *Cbp53E* |
| *CG8249* | *Fic* | *CG32206* | *Rfx* | *FR* | *Cortactin* | *Nak* |
| *Tsf3* | *scylla* | *Hs3st-A* | *Mgat2* | *CG9304* | *Fas1* | *Tkr* |
| *CG13043* | *ico* | *PR-Set7* | *emp* | *CG16886* | *Fhos* | *CG12344* |
| *CG5608* | *CG4462* | *Acp1* | *CG43780* | *ERR* | *Gyc76C* | *CG43729* |

**Table S5. Networks associated GO biological functions**. GO Biological functions represented (+) in the interaction groups for each of the 3 arrays experiments (Diapause, Photoperiodic and miRNA)

| **Biological functions** | **Diapause** | **Photoperiodic** | **miRNA** |
|---|---|---|---|
| Olfactory Behavior | + | + | + |
| Oxidation-reduction | + | + | + |
| Courtship behaviour | + | + | + |
| Fatty acid biosynthetic process | + | + | + |
| Regulation of alternative nuclear mRNA splicing | + | + | + |
| Chromatin silencing | + | + | + |
| Regulation of Notch signaling pathway | + | + | + |
| Aggressive behaviour | + | + | + |
| Neurotransmitter secretion | + | + | + |
| Histone methylation | + | + | + |
| Long-term memory | + | + | + |
| Determination of adult lifespan | + | + | + |
| Phototransduction | + | + | + |
| Carbohydrate metabolic process | + | + | + |
| Apoptosis | + | + | + |
| Diapause | - | + | - |
| rRNA methylation | - | + | - |
| Histone phosphorylation | - | + | - |
| Gene silencing by miRNA | - | + | - |
| Regulation of circadian sleep/wake cycle | - | + | - |
| Entrainment of circadian clock | - | + | + |
| Regulation of circadian rhythm | - | + | + |
| Response to heat | - | + | + |
| Visual behavior | - | + | + |
| JAK-STAT cascade | - | + | + |

| | | | |
|---|---|---|---|
| Fat body development | + | - | - |
| tRNA methylation | + | + | - |
| Regulation of translational initiation | + | + | - |
| Phospholipid biosynthetic process | + | + | - |
| Ecdysone receptor-mediated signaling pathway | + | - | + |
| Positive regulation of hormone secretion | + | - | + |
| Rhodopsin mediated signaling pathway | + | - | + |
| Mitochondrial electron transport | - | - | + |

**Table S6 AGO-1 Immunoprecipitation Enriched transcripts**

| Long day Enriched transcripts | | | | | | | |
|---|---|---|---|---|---|---|---|
| **FlyBaseID** | **Gene** | **FlyBaseID** | **Gene** | **FlyBaseID** | **Gene** | **FlyBaseID** | **Gene** |
| *FBgn0040297* | *Nhe2* | *FBgn0052137* | *CG32137* | *FBgn0035087* | *CG2765* | *FBgn0020513* | *ade5* |
| *FBgn0000479* | *dnc* | *FBgn0285926* | *Imp* | *FBgn0028292* | *ric8a* | *FBgn0028863* | *CG4587* |
| *FBgn0261041* | *stj* | *FBgn0034389* | *Mctp* | *FBgn0001404* | *egh* | *FBgn0085422* | *CG34393* |
| *FBgn0262739* | *AGO1* | *FBgn0263993* | *CG43736* | *FBgn0031791* | *AANATL2* | *FBgn0026160* | *tna* |
| *FBgn0031090* | *Rab35* | *FBgn0031681* | *pgant5* | *FBgn0034136* | *DAT* | *FBgn0086779* | *step* |
| *FBgn0085408* | *Shroom* | *FBgn0036202* | *CG6024* | *FBgn0031896* | *CG4502* | *FBgn0062440* | *CG17680* |
| *FBgn0038740* | *CG4562* | *FBgn0035888* | *CG7120* | *FBgn0001320* | *kni* | *FBgn0030291* | *CG1738* |
| *FBgn0041160* | *comm2* | *FBgn0265630* | *sno* | *FBgn0026597* | *Axn* | *FBgn0262738* | *norpA* |
| *FBgn0030520* | *Pdcd4* | *FBgn0085390* | *Dgk* | *FBgn0032886* | *CG9328* | *FBgn0039644* | *CG11897* |
| *FBgn0003326* | *sca* | *FBgn0021764* | *sdk* | *FBgn0001229* | *Hsp67Bc* | *FBgn0262743* | *Fs(2)Ket* |
| *FBgn0033438* | *Mmp2* | *FBgn0035558* | *CG11357* | *FBgn0027596* | *Kank* | *FBgn0085447* | *sif* |
| *FBgn0026263* | *bip1* | *FBgn0029837* | *Tsp5D* | *FBgn0024734* | *PRL-1* | *FBgn0039151* | *CG13607* |
| *FBgn0083972* | *CG34136* | *FBgn0030503* | *Tango2* | *FBgn0086707* | *ncm* | *FBgn0000964* | *tj* |
| *FBgn0033936* | *CG17386* | *FBgn0037956* | *CG6959* | *FBgn0040375* | *CG13760* | *FBgn0024921* | *Trn* |
| *FBgn0036104* | *CG6418* | *FBgn0026206* | *mei-P26* | *FBgn0003091* | *Pkc53E* | *FBgn0032763* | *CG17568* |
| *FBgn0004606* | *zfh1* | *FBgn0035986* | *CG4022* | *FBgn0051324* | *CG31324* | *FBgn0028708* | *Mst85C* |
| *FBgn0030482* | *CG1673* | *FBgn0033638* | *CG9005* | *FBgn0029878* | *Pat1* | *FBgn0283741* | *prage* |
| *FBgn0036360* | *CG10713* | *FBgn0029666* | *CG10803* | *FBgn0032957* | *CG2225* | *FBgn0029810* | *CG12239* |
| *FBgn0039459* | *IntS12* | *FBgn0261963* | *mid* | *FBgn0033761* | *CG8778* | *FBgn0042083* | *CG3267* |
| *FBgn0002939* | *ninaD* | *FBgn0004635* | *rho* | *FBgn0263106* | *DnaJ-1* | *FBgn0029903* | *pod1* |
| *FBgn0037022* | *CG11396* | *FBgn0014133* | *bif* | *FBgn0010316* | *dap* | *FBgn0261673* | *nemy* |
| *FBgn0004611* | *Plc21C* | *FBgn0000179* | *bi* | *FBgn0015799* | *Rbf* | *FBgn0039688* | *Kul* |
| *FBgn0039013* | *CG4813* | *FBgn0039329* | *CG10669* | *FBgn0052767* | *CG32767* | *FBgn0004903* | *Rb97D* |
| *FBgn0029676* | *HIP-R* | *FBgn0266450* | *Kr-h1* | *FBgn0033486* | *dmpd* | *FBgn0037130* | *Syn1* |
| *FBgn0004198* | *ct* | *FBgn0036059* | *nudE* | *FBgn0050158* | *CG30158* | *FBgn0015296* | *Shc* |
| *FBgn0003525* | *stg* | *FBgn0033520* | *Prx2540-1* | *FBgn0040609* | *CG3348* | *FBgn0029887* | *CG3198* |

| | | | | | | | |
|---|---|---|---|---|---|---|---|
| *FBgn0034210* | *CG6568* | *FBgn0029935* | *CG4615* | *FBgn0039325* | *CG10560* | *FBgn0022936* | *CycH* |
| *FBgn0033639* | *CG9003* | *FBgn0030208* | *PPP4R2r* | *FBgn0031424* | *VGlut* | *FBgn0017590* | *klg* |
| *FBgn0029003* | *mab-21* | *FBgn0003444* | *smo* | *FBgn0027342* | *fz4* | *FBgn0032717* | *CG10600* |
| *FBgn0035762* | *Rint1* | *FBgn0050000* | *GstT1* | *FBgn0266101* | *CG44838* | *FBgn0038815* | *CG5466* |
| *FBgn0039734* | *Tace* | *FBgn0036208* | *CG10361* | *FBgn0010113* | *hdc* | *FBgn0004369* | *Ptp99A* |
| *FBgn0264672* | *Eogt* | *FBgn0026573* | *ADD1* | *FBgn0264848* | *vih* | *FBgn0086358* | *Tab2* |
| *FBgn0085409* | *smal* | *FBgn0030505* | *NFAT* | *FBgn0034081* | *CG10731* | *FBgn0036341* | *Syx13* |
| *FBgn0038870* | *Oga* | *FBgn0036155* | *CG6163* | *FBgn0033984* | *Lap1* | *FBgn0040505* | *Alk* |
| *FBgn0032233* | *dpr19* | *FBgn0039266* | *CG11791* | *FBgn0261611* | *CG42700* | *FBgn0029761* | *SK* |

**Long day Enriched transcripts**

| **FlyBaseID** | **Gene** | **FlyBaseID** | **Gene** | **FlyBaseID** | **Gene** | **FlyBaseID** | **Gene** |
|---|---|---|---|---|---|---|---|
| *FBgn0039505* | *CG5934* | *FBgn0003396* | *shn* | *FBgn0027583* | *CG7601* | *FBgn0030037* | *Miga* |
| *FBgn0031375* | *erm* | *FBgn0011818* | *oaf* | *FBgn0044323* | *Cka* | *FBgn0040396* | *CG3939* |
| *FBgn0030467* | *CG1764* | *FBgn0035625* | *Blimp-1* | *FBgn0036398* | *upSET* | *FBgn0034091* | *mrj* |
| *FBgn0039089* | *beat-IV* | *FBgn0039487* | *gb* | *FBgn0015400* | *kek2* | *FBgn0035186* | *CG13912* |
| *FBgn0013997* | *Nrx-IV* | *FBgn0039789* | *CG9717* | *FBgn0023526* | *CG2865* | *FBgn0035674* | *CG13295* |
| *FBgn0011817* | *nmo* | *FBgn0038725* | *CG6184* | *FBgn0029939* | *CG9650* | *FBgn0039311* | *CG10513* |
| *FBgn0267821* | *da* | *FBgn0029123* | *SoxN* | *FBgn0261239* | *Hr39* | *FBgn0027504* | *CG8878* |
| *FBgn0038043* | *CG17202* | *FBgn0036196* | *CG11658* | *FBgn0033313* | *Cirl* | *FBgn0033117* | *CG3358* |
| *FBgn0262740* | *Evi5* | *FBgn0260400* | *elav* | *FBgn0031698* | *Ncoa6* | *FBgn0033879* | *CG6543* |
| *FBgn0263027* | *CG43322* | *FBgn0004652* | *fru* | *FBgn0005775* | *Con* | *FBgn0034032* | *CG8195* |
| *FBgn0263026* | *CG43321* | *FBgn0030228* | *BTBD9* | *FBgn0023528* | *CG2924* | *FBgn0027539* | *lili* |
| *FBgn0035914* | *CG6282* | *FBgn0033915* | *CG8485* | *FBgn0051300* | *CG31300* | *FBgn0010452* | *trn* |
| *FBgn0050389* | *CG30389* | *FBgn0051221* | *CG31221* | *FBgn0259736* | *CG42390* | *FBgn0031319* | *CG4896* |
| *FBgn0001235* | *hth* | *FBgn0033544* | *CG7220* | *FBgn0085450* | *Snoo* | *FBgn0263240* | *Coop* |
| *FBgn0004595* | *pros* | *FBgn0037817* | *Cyp12e1* | *FBgn0043364* | *cbt* | *FBgn0263772* | *CG43689* |
| *FBgn0023215* | *Mnt* | *FBgn0051163* | *SKIP* | *FBgn0029504* | *CHES-1-like* | *FBgn0030725* | *CG8958* |

| | | | | | | | |
|---|---|---|---|---|---|---|---|
| *FBgn0053147* | *Hs3st-A* | *FBgn0036165* | *chrb* | *FBgn0025702* | *Srpk79D* | *FBgn0052432* | *CG32432* |
| *FBgn0259984* | *kuz* | *FBgn0003209* | *raw* | *FBgn0028582* | *lqf* | *FBgn0264542* | *hwt* |
| *FBgn0032723* | *ssp3* | *FBgn0017581* | *Lk6* | *FBgn0261986* | *RASSF8* | *FBgn0040075* | *rept* |
| *FBgn0063497* | *GstE3* | *FBgn0036249* | *CG11560* | *FBgn0032901* | *sky* | *FBgn0033654* | *Sobp* |
| *FBgn0039283* | *danr* | *FBgn0031745* | *rau* | *FBgn0037636* | *CG9821* | *FBgn0033518* | *Prx2540-2* |
| *FBgn0016076* | *vri* | *FBgn0250908* | *beat-VII* | *FBgn0035719* | *tow* | *FBgn0031398* | *CG10880* |
| *FBgn0263511* | *Vsx1* | *FBgn0031939* | *CG13796* | *FBgn0267488* | *Mcr* | *FBgn0033784* | *SCCRO3* |
| *FBgn0261954* | *east* | *FBgn0032949* | *Lamp1* | *FBgn0001230* | *Hsp68* | *FBgn0004811* | *fs(2)ltoPP43* |
| *FBgn0263846* | *CG43707* | *FBgn0033649* | *pyr* | *FBgn0004168* | *5-HT1A* | *FBgn0004396* | *CrebA* |
| *FBgn0262476* | *CG43066* | *FBgn0003386* | *Shaw* | *FBgn0039696* | *CG7837* | *FBgn0040336* | *Seipin* |
| *FBgn0033474* | *CG1407* | *FBgn0033935* | *Sin1* | *FBgn0038828* | *CG17270* | *FBgn0003520* | *stau* |
| *FBgn0039223* | *CG5805* | *FBgn0037849* | *CG4596* | *FBgn0261560* | *Thor* | *FBgn0025809* | *Paf-AHalpha* |
| *FBgn0031738* | *CG9171* | *FBgn0039113* | *CG10217* | *FBgn0051092* | *LpR2* | *FBgn0032946* | *nrv3* |
| *FBgn0000411* | *D* | *FBgn0000492* | *Dr* | *FBgn0036264* | *CG11529* | *FBgn0040305* | *MTF-1* |
| *FBgn0016078* | *wun* | *FBgn0031397* | *CG15385* | *FBgn0003218* | *rdgB* | *FBgn0030090* | *fend* |
| *FBgn0030276* | *Dlic* | *FBgn0035907* | *GstO1* | *FBgn0027506* | *EDTP* | *FBgn0010105* | *comm* |
| *FBgn0039075* | *CG4393* | *FBgn0010300* | *brat* | *FBgn0051710* | *CG31710* | *FBgn0039663* | *CG2321* |
| *FBgn0035975* | *PGRP-LA* | *FBgn0033153* | *Gadd45* | *FBgn0011591* | *fng* | *FBgn0033901* | *O-fut1* |
| *FBgn0264307* | *orb2* | *FBgn0010482* | *l(2)01289* | *FBgn0085391* | *trv* | *FBgn0004244* | *Rdl* |

**Long day Enriched transcripts**

| FlyBaseID | Gene | FlyBaseID | Gene | FlyBaseID | Gene | FlyBaseID | Gene |
|---|---|---|---|---|---|---|---|
| *FBgn0031697* | *CG14024* | *FBgn0031461* | *daw* | *FBgn0011705* | *rost* | *FBgn0033911* | *VGAT* |
| *FBgn0015838* | *Vang* | *FBgn0264962* | *Pcf11* | *FBgn0011746* | *ana* | *FBgn0001291* | *Jra* |
| *FBgn0010238* | *Lac* | *FBgn0266411* | *sima* | *FBgn0001206* | *Hmr* | *FBgn0014073* | *Tie* |
| *FBgn0003067* | *Pepck* | *FBgn0261383* | *IntS6* | *FBgn0262473* | *Tl* | *FBgn0033730* | *Cpr49Ag* |
| *FBgn0032730* | *CG10431* | *FBgn0283680* | *IP3K2* | *FBgn0039532* | *Mtl* | *FBgn0039507* | *mrt* |
| *FBgn0086655* | *jing* | *FBgn0004512* | *Mdr49* | *FBgn0261555* | *CG42673* | *FBgn0086677* | *jeb* |

| | | | | | | | |
|---|---|---|---|---|---|---|---|
| *FBgn0000546* | *EcR* | *FBgn0039419* | *CG12290* | *FBgn0005586* | *Rab3* | *FBgn0031399* | *mio* |
| *FBgn0031457* | *CG3077* | *FBgn0283472* | *S6k* | *FBgn0052380* | *SMSr* | *FBgn0026319* | *Traf4* |
| *FBgn0037140* | *SLC22A* | *FBgn0267253* | *CG32700* | *FBgn0031558* | *CG16704* | *FBgn0023388* | *Dap160* |
| *FBgn0035688* | *CG10289* | *FBgn0023076* | *Clk* | *FBgn0030529* | *Clic* | *FBgn0033087* | *Hsepi* |
| *FBgn0267001* | *Ten-a* | *FBgn0034223* | *Tes* | *FBgn0031359* | *Rim2* | *FBgn0035802* | *Pura* |
| *FBgn0263930* | *dally* | *FBgn0016694* | *Pdp1* | *FBgn0003053* | *peb* | *FBgn0011666* | *msi* |
| *FBgn0034390* | *CG15093* | *FBgn0029828* | *CG6067* | *FBgn0036519* | *CG7650* | *FBgn0003028* | *ovo* |
| *FBgn0033391* | *CG8026* | *FBgn0085423* | *CG34394* | *FBgn0004509* | *Fur1* | *FBgn0085383* | *CG34354* |
| *FBgn0003093* | *Pkc98E* | *FBgn0013765* | *cnn* | *FBgn0001084* | *fy* | *FBgn0034417* | *CG15117* |
| *FBgn0039994* | *conu* | *FBgn0000464* | *Lar* | *FBgn0243513* | *cnir* | *FBgn0024230* | *Hs2st* |
| *FBgn0002932* | *neur* | *FBgn0039584* | *beat-VI* | *FBgn0034331* | *CG15067* | *FBgn0037848* | *Tsp86D* |
| *FBgn0264502* | *CG43901* | *FBgn0010894* | *sinu* | *FBgn0039380* | *CG5890* | *FBgn0003044* | *Pcl* |
| *FBgn0039431* | *plum* | *FBgn0053639* | *CG33639* | *FBgn0029768* | *SPR* | *FBgn0010303* | *hep* |
| *FBgn0034265* | *Snx16* | *FBgn0263352* | *Unr* | *FBgn0020767* | *Spred* | *FBgn0040636* | *CG13255* |
| *FBgn0263773* | *fok* | *FBgn0000382* | *csw* | *FBgn0000119* | *arr* | *FBgn0029778* | *RhoGAP5A* |
| *FBgn0029522* | *CG13373* | *FBgn0024315* | *Picot* | *FBgn0086675* | *fne* | *FBgn0022800* | *Cad96Ca* |
| *FBgn0263005* | *CG43313* | *FBgn0015000* | *betaggt-I* | *FBgn0000636* | *Fas3* | *FBgn0037906* | *PGRP-LB* |
| *FBgn0010473* | *tutl* | *FBgn0265276* | *l(3)neo38* | *FBgn0039066* | *EloA* | *FBgn0036043* | *CG8177* |
| *FBgn0086758* | *chinmo* | *FBgn0032036* | *CG13384* | *FBgn0037106* | *CG11307* | *FBgn0013548* | *l(2)dtl* |
| *FBgn0265605* | *Ric* | *FBgn0033791* | *Drl-2* | *FBgn0015229* | *glec* | *FBgn0053145* | *GalT1* |
| *FBgn0042111* | *CG18766* | *FBgn0085369* | *Drgx* | *FBgn0015828* | *TfIIEalpha* | *FBgn0050080* | *CG30080* |
| *FBgn0000043* | *Act42A* | *FBgn0031969* | *pes* | *FBgn0032078* | *C1GalTA* | *FBgn0261279* | *lqfR* |
| *FBgn0013732* | *sced* | *FBgn0030530* | *jub* | *FBgn0002643* | *mam* | *FBgn0027561* | *CG18659* |
| *FBgn0036299* | *Tsf2* | *FBgn0032042* | *CG13398* | *FBgn0053547* | *Rim* | *FBgn0031703* | *CG12512* |
| *FBgn0066114* | *GlcAT-I* | *FBgn0038946* | *rdhB* | *FBgn0011206* | *bol* | *FBgn0260945* | *Atg1* |
| *FBgn0005616* | *msl-2* | *FBgn0035656* | *CG10479* | *FBgn0264495* | *gpp* | *FBgn0014011* | *Rac2* |
| *FBgn0035959* | *CG4911* | *FBgn0002723* | *Met* | *FBgn0260657* | *CG42540* | *FBgn0283499* | *InR* |
| *FBgn0024294* | *Spn43Aa* | *FBgn0036449* | *bmm* | *FBgn0085382* | *CG34353* | *FBgn0030294* | *Pal* |
| *FBgn0033521* | *CG12896* | *FBgn0083961* | *CG34125* | *FBgn0259150* | *CG42265* | *FBgn0001297* | *kay* |

| Long day Enriched transcripts | | | | | | | |
|---|---|---|---|---|---|---|---|
| **FlyBaseID** | **Gene** | **FlyBaseID** | **Gene** | **FlyBaseID** | **Gene** | **FlyBaseID** | **Gene** |
| *FBgn0266000* | *CG44774* | *FBgn0086365* | *Orct2* | *FBgn0041723* | *rho-5* | *FBgn0040153* | *l(1)G0469* |
| *FBgn0033782* | *sug* | *FBgn0003302* | *rux* | *FBgn0283712* | *LIMK1* | *FBgn0261823* | *Asx* |
| *FBgn0016977* | *spen* | *FBgn0262730* | *dtn* | *FBgn0037853* | *CG14696* | *FBgn0040765* | *luna* |
| *FBgn0039254* | *Nmnat* | *FBgn0038821* | *CG17267* | *FBgn0030089* | *AP-1gamma* | *FBgn0030260* | *CG1537* |
| *FBgn0031988* | *CG8668* | *FBgn0000659* | *fkh* | *FBgn0034184* | *CG9646* | *FBgn0034181* | *CG8963* |
| *FBgn0034070* | *SP2353* | *FBgn0052423* | *shep* | *FBgn0051158* | *Efa6* | *FBgn0036259* | *CG9760* |
| *FBgn0000490* | *dpp* | *FBgn0000097* | *aop* | *FBgn0039461* | *CG5500* | *FBgn0085407* | *Pvf3* |
| *FBgn0036198* | *crim* | *FBgn0038818* | *Nep4* | *FBgn0039647* | *CG14509* | *FBgn0011224* | *heph* |
| *FBgn0025633* | *CG13366* | *FBgn0036374* | *Spt20* | *FBgn0036099* | *CG11811* | *FBgn0015618* | *Cdk8* |
| *FBgn0000547* | *ed* | *FBgn0031904* | *CG5149* | *FBgn0051116* | *ClC-a* | *FBgn0027532* | *CG7139* |
| *FBgn0033497* | *CG12912* | *FBgn0031769* | *CG9135* | *FBgn0031817* | *CG9531* | *FBgn0001227* | *Hsp67Ba* |
| *FBgn0026582* | *Hmg-2* | *FBgn0039244* | *CG11069* | *FBgn0005672* | *spi* | *FBgn0034300* | *CG5098* |
| *FBgn0033872* | *CG6329* | *FBgn0030156* | *CG15247* | *FBgn0011837* | *Tis11* | *FBgn0005427* | *ewg* |
| *FBgn0033261* | *udd* | *FBgn0029914* | *CG4558* | *FBgn0033250* | *CG14762* | *FBgn0040942* | *CG12643* |
| *FBgn0032026* | *CG7627* | *FBgn0003415* | *skd* | *FBgn0267348* | *LanB2* | *FBgn0030114* | *CG17754* |
| *FBgn0038730* | *CG6300* | *FBgn0028394* | *CG17834* | *FBgn0259745* | *wech* | *FBgn0264273* | *Sema2b* |
| *FBgn0031992* | *CG8498* | *FBgn0085420* | *DIP-delta* | *FBgn0039178* | *CG6356* | *FBgn0040817* | *CG14132* |
| *FBgn0266409* | *CG45049* | *FBgn0029905* | *Nf-YC* | *FBgn0037057* | *CG10512* | *FBgn0263352* | *Unr* |
| *FBgn0016070* | *smg* | *FBgn0033715* | *CG8490* | *FBgn0038975* | *Nrx-1* | *FBgn0264303* | *CG43781* |
| *FBgn0283521* | *lola* | *FBgn0035397* | *CG11486* | *FBgn0053512* | *dpr4* | *FBgn0264302* | *CG43780* |
| *FBgn0030522* | *CG11103* | *FBgn0052091* | *CG32091* | *FBgn0037814* | *CG6325* | *FBgn0029118* | *Sucb* |
| *FBgn0038053* | *CG18549* | *FBgn0031881* | *MME1* | *FBgn0033988* | *pcs* | *FBgn0039186* | *CG5746* |
| *FBgn0051774* | *fred* | *FBgn0259111* | *Ndae1* | *FBgn0037999* | *CG4860* | *FBgn0037960* | *mthl5* |
| *FBgn0011300* | *babo* | *FBgn0053193* | *sav* | *FBgn0044047* | *Ilp6* | *FBgn0262579* | *Ect4* |
| *FBgn0038853* | *RhoGAP93B* | *FBgn0052369* | *CG32369* | *FBgn0029711* | *Usf* | *FBgn0033032* | *kune* |
| *FBgn0039029* | *CG4704* | *FBgn0010313* | *corto* | *FBgn0037107* | *CG7166* | *FBgn0030634* | *CG9164* |

| | | | | | | | |
|---|---|---|---|---|---|---|---|
| *FBgn0034394* | *CG15096* | *FBgn0037852* | *Tpc1* | *FBgn0030183* | *CG15309* | *FBgn0261989* | *CG42807* |
| *FBgn0015399* | *kek1* | *FBgn0033799* | *GLaz* | *FBgn0265935* | *coro* | *FBgn0261990* | *CG42808* |
| *FBgn0039277* | *CG13650* | *FBgn0032629* | *beat-IIIc* | *FBgn0033656* | *S2P* | *FBgn0015380* | *drl* |
| *FBgn0263316* | *Mrp4* | *FBgn0004861* | *ph-p* | *FBgn0001137* | *grk* | *FBgn0035617* | *l(3)psg2* |
| *FBgn0053110* | *CG33110* | *FBgn0028743* | *Dhit* | *FBgn0283680* | *IP3K2* | *FBgn0031882* | *Rab30* |
| *FBgn0038326* | *CG5044* | *FBgn0028496* | *CG30116* | *FBgn0031835* | *CG11319* | *FBgn0038862* | *Usp8* |
| *FBgn0023214* | *edl* | *FBgn0000459* | *disco* | *FBgn0267792* | *rgr* | *FBgn0013718* | *nuf* |
| *FBgn0034191* | *CG6984* | *FBgn0033010* | *Atf6* | *FBgn0028683* | *spt4* | *FBgn0033677* | *CG8321* |
| *FBgn0261258* | *rgn* | *FBgn0260499* | *qvr* | *FBgn0039966* | *Rab21* | *FBgn0036152* | *CG6175* |

| **Long day Enriched transcripts** | | | | | |
|---|---|---|---|---|---|
| **FlyBaseID** | **Gene** | **FlyBaseID** | **Gene** | **FlyBaseID** | **Gene** |
| *FBgn0050431* | *CG30431* | *FBgn0040827* | *CG13315* | *UNK* | *rtGEF* |
| *FBgn0030049* | *Trf4-1* | *FBgn0265195* | *veg* | *UNK* | *CG31108* |
| *FBgn0039492* | *CG6051* | *FBgn0014343* | *mirr* | *UNK* | *rap* |
| *FBgn0004893* | *bowl* | *FBgn0035676* | *ssp6* | *UNK* | *Gef26* |
| *FBgn0029974* | *dpr14* | *UNK* | *Mpk2* | *UNK* | *CG18327* |
| *FBgn0261437* | *CSN8* | *UNK* | *AIF,CG7261* | *UNK* | *DLP* |
| *FBgn0259246* | *brp* | *UNK* | *Nc* | *UNK* | *CG32830,ab* |
| *FBgn0261617* | *nej* | *UNK* | *lea* | *UNK* | *mp* |
| *FBgn0015522* | *olf186-M* | *UNK* | *neuroligin* | *UNK* | *Drep-2* |
| *FBgn0003460* | *so* | *UNK* | *Utx,trk* | *UNK* | *Sug* |
| *FBgn0032105* | *borr* | *UNK* | *Ice* | *UNK* | *Arp87C* |
| *FBgn0267033* | *mamo* | *UNK* | *Kdm4B,seq* | *UNK* | *Mdh* |
| *FBgn0039719* | *CG15515* | *UNK* | *Sema-2a* | *UNK* | *ia2* |
| *FBgn0259234* | *Camta* | *UNK* | *l(1)G0232* | | |
| *FBgn0265595* | *CG44422* | *UNK* | *iHog* | | |
| *FBgn0041094* | *scyl* | *UNK* | *Gap1* | | |

| | | | |
|---|---|---|---|
| *FBgn0030010* | *CG10959* | *UNK* | *CG15742,mew* |
| *FBgn0086676* | *spin* | *UNK* | *CG34280,Sur-8* |
| *FBgn0033075* | *Pld* | *UNK* | *tomosyn* |
| *FBgn0028978* | *trbl* | *UNK* | *nAcRalpha-96Aa* |
| *FBgn0031384* | *CG4238* | *UNK* | *tankyrase* |
| *FBgn0037016* | *CG13252* | *UNK* | *Sema-1b* |
| *FBgn0029663* | *CG10804* | *UNK* | *yu* |
| *FBgn0036194* | *CG11652* | *UNK* | *l(1)G0148* |
| *FBgn0020622* | *Pi3K21B* | *UNK* | *l(2)k16918* |
| *FBgn0029932* | *CG4607* | *UNK* | *Zip3* |
| *FBgn0261793* | *Trf2* | *UNK* | *dm* |
| *FBgn0000352* | *cos* | *UNK* | *Caki* |
| *FBgn0025373* | *Fpps* | *UNK* | *E2f* |
| *FBgn0001168* | *h* | *UNK* | *tan* |
| *FBgn0036372* | *Abp1* | *UNK* | *Usp36* |
| *FBgn0052062* | *Rbfox1* | *UNK* | *CG31697,CG33116* |
| *FBgn0033031* | *CG8245* | *UNK* | *l(2)08717* |
| *FBgn0039465* | *Tsp97E* | *UNK* | *nAcRalpha-34E* |
| *FBgn0034304* | *CG5742* | *UNK* | *Sema-5c* |

**Short day Enriched transcripts**

| FlyBaseID | Gene | FlyBaseID | Gene | FlyBaseID | Gene |
|---|---|---|---|---|---|
| *FBgn0040153* | *l(1)G0469* | *FBgn0283499* | *InR* | *FBgn0264962* | *Pcf11* |
| *FBgn0039994* | *conu* | *FBgn0053639* | *CG33639* | *FBgn0032957* | *CG2225* |
| *FBgn0035916* | *GAPsec* | *FBgn0015000* | *betaggt-I* | *FBgn0283680* | *IP3K2* |
| *FBgn0040765* | *luna* | *FBgn0028743* | *Dhit* | *FBgn0035625* | *Blimp-1* |
| *FBgn0015541* | *sda* | *FBgn0031969* | *pes* | *FBgn0263106* | *DnaJ-1* |
| *FBgn0030260* | *CG1537* | *FBgn0033983* | *ADPS* | *FBgn0027342* | *fz4* |

| | | | | | |
|---|---|---|---|---|---|
| *FBgn0031896* | *CG4502* | *FBgn0004893* | *bowl* | *FBgn0037906* | *PGRP-LB* |
| *FBgn0001320* | *kni* | *FBgn0035558* | *CG11357* | *FBgn0031882* | *Rab30* |
| *FBgn0032782* | *Rab9* | *FBgn0266000* | *CG44774* | *FBgn0033677* | *CG8321* |
| *FBgn0261800* | *LanB1* | *FBgn0038946* | *rdhB* | *FBgn0028467* | *CG11070* |
| *FBgn0020312* | *CG4050* | *FBgn0025809* | *Paf-AHalpha* | *FBgn0053494* | *CG33494* |
| *FBgn0000043* | *Act42A* | *FBgn0003460* | *so* | *FBgn0052432* | *CG32432* |
| *FBgn0026263* | *bip1* | *FBgn0039151* | *CG13607* | *FBgn0028863* | *CG4587* |
| *FBgn0031589* | *CG3714* | *FBgn0011746* | *ana* | *FBgn0010313* | *corto* |
| *FBgn0040375* | *CG13760* | *FBgn0036198* | *crim* | *FBgn0010894* | *sinu* |
| *FBgn0040942* | *CG12643* | *FBgn0033698* | *CG8858* | *FBgn0030174* | *CG15312* |
| *FBgn0016078* | *wun* | *FBgn0010482* | *l(2)01289* | *FBgn0001168* | *h* |
| *FBgn0035959* | *CG4911* | *FBgn0031384* | *CG4238* | *FBgn0037057* | *CG10512* |
| *FBgn0028394* | *CG17834* | *FBgn0032763* | *CG17568* | *FBgn0015838* | *Vang* |
| *FBgn0284256* | *bsf* | *FBgn0034480* | *CG16898* | *UNK* | *dm* |
| *FBgn0033031* | *CG8245* | *FBgn0001234* | *Hsromega* | *UNK* | *AIF,CG7261* |
| *FBgn0050000* | *GstT1* | *FBgn0043364* | *cbt* | *UNK* | *Drep-2* |
| *FBgn0039029* | *CG4704* | *FBgn0004635* | *rho* | | |

**Table S7.** Long day and short day immunoprecipitation common enriched genes

| Common Enriched Genes | | | | | |
|---|---|---|---|---|---|
| **FlyBaseID** | **Gene** | **FlyBaseID** | **Gene** | **FlyBaseID** | **Gene** |
| *FBgn0026263* | *bip1* | *FBgn0039029* | *CG4704* | *FBgn0039994* | *conu* |
| *FBgn0035558* | *CG11357* | *FBgn0028394* | *CG17834* | *FBgn0000043* | *Act42A* |
| *FBgn0004635* | *rho* | *FBgn0010313* | *corto* | *FBgn0035959* | *CG4911* |
| *FBgn0050000* | *GstT1* | *FBgn0028743* | *Dhit* | *FBgn0264962* | *Pcf11* |
| *FBgn0031896* | *CG4502* | *FBgn0037057* | *CG10512* | *FBgn0010894* | *sinu* |
| *FBgn0001320* | *kni* | *FBgn0283680* | *IP3K2* | *FBgn0266000* | *CG44774* |
| *FBgn0040375* | *CG13760* | *FBgn0040153* | *l(1)G0469* | *FBgn0036198* | *crim* |
| *FBgn0032957* | *CG2225* | *FBgn0040765* | *luna* | *FBgn0053639* | *CG33639* |
| *FBgn0263106* | *DnaJ-1* | *FBgn0030260* | *CG1537* | *FBgn0015000* | *betaggt-I* |
| *FBgn0027342* | *fz4* | *FBgn0040942* | *CG12643* | *FBgn0031969* | *pes* |
| *FBgn0028863* | *CG4587* | *FBgn0031882* | *Rab30* | *FBgn0038946* | *rdhB* |
| *FBgn0039151* | *CG13607* | *FBgn0033677* | *CG8321* | *FBgn0025809* | *Paf-AHalpha* |
| *FBgn0032763* | *CG17568* | *FBgn0004893* | *bowl* | *FBgn0015838* | *Vang* |
| *FBgn0016078* | *wun* | *FBgn0003460* | *so* | *FBgn0037906* | *PGRP-LB* |
| *FBgn0035625* | *Blimp-1* | *FBgn0031384* | *CG4238* | *FBgn0283499* | *InR* |
| *FBgn0010482* | *l(2)01289* | *FBgn0001168* | *h* | *FBgn0011746* | *ana* |
| *FBgn0043364* | *cbt* | *FBgn0033031* | *CG8245* | *FBgn0052432* | *CG32432* |

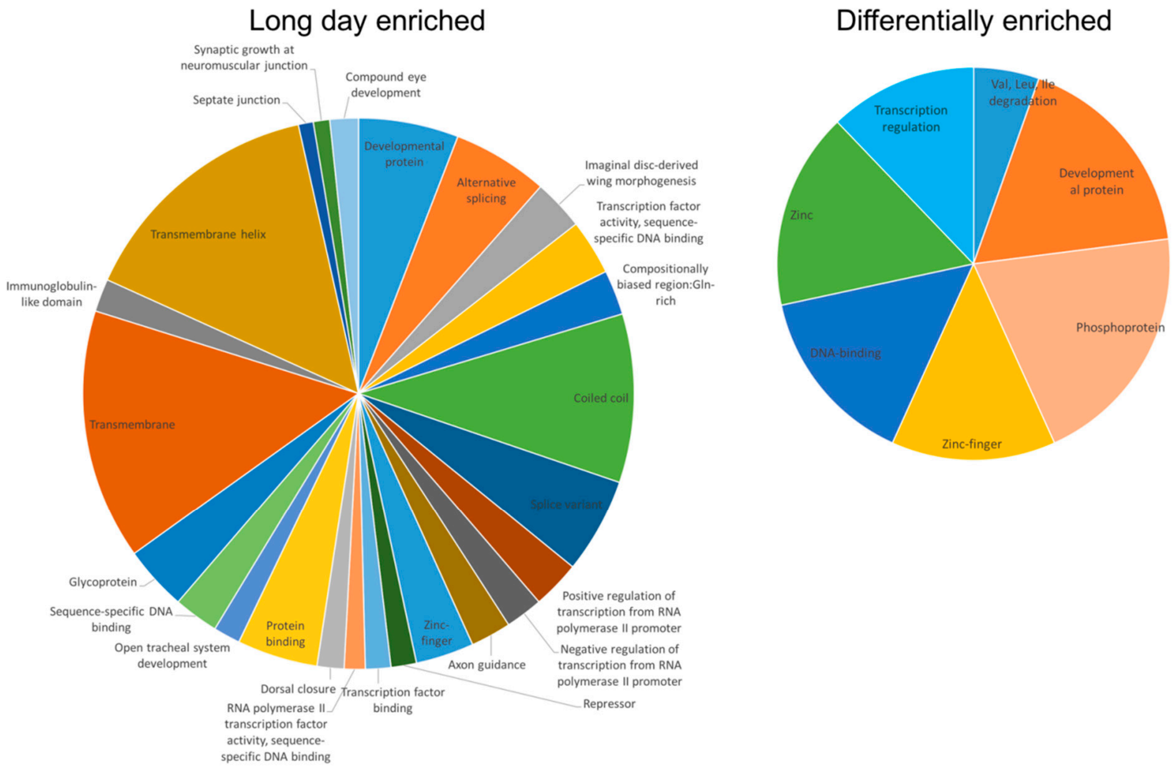


**Figure S1.** Enriched biological functions among AGO-1 immunoprecipitation transcripts. Pie chart representing the 25 most significant enriched biological functions (DAVID, Benjamini $p<0.01$) of the enriched gene transcripts in long day (left). Pie chart representing significant enriched functions (DAVID, Benjamini $p<0.05$) of the differentially enriched genes between long and short day (right). The size of the sections is proportional to the number of genes in the enriched biological function.

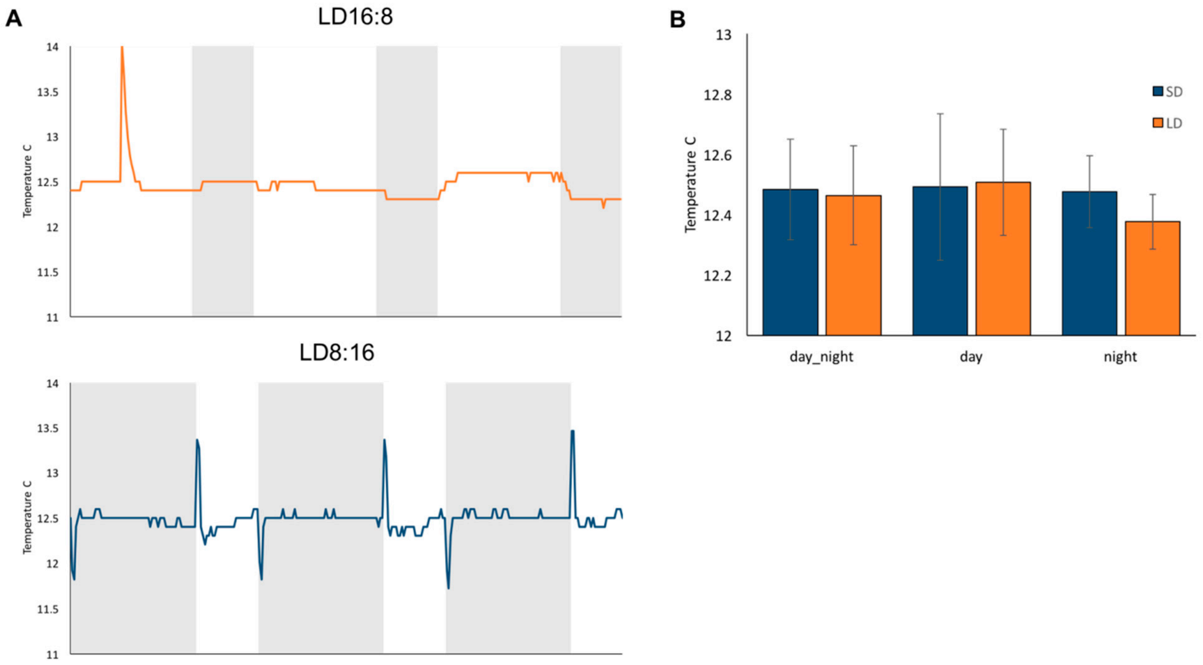


**Figure S2. Temperature measurements in the light boxes**. **A.** Three days temperature traces during the long 16:8 (top) or the short 8:16 (bottom) photoperiods. Grey shadows represent nights. **B.** Average temperature differences between long (12.46 ± 0.16 ºC, average ± SD) and short (12.48 ± 0.17 ºC) day boxes. Difference in temperature is also compared for days only (long photoperiod 12.51 ± 0.18 ºC; short photoperiod 12.49 ± 0.24 ºC) and for nights only (long photoperiod 12.38 ± 0.09 ºC; short photoperiod 12.48 ± 0.12 ºC).
